# RA and FGF Signalling Are Required in the Zebrafish Otic Vesicle to Pattern and Maintain Ventral Otic Identities

**DOI:** 10.1371/journal.pgen.1004858

**Published:** 2014-12-04

**Authors:** Esther C. Maier, Tanya T. Whitfield

**Affiliations:** MRC Centre for Developmental and Biomedical Genetics, Bateson Centre and Department of Biomedical Science, University of Sheffield, Sheffield, United Kingdom; University of Pennsylvania School of Medicine, United States of America

## Abstract

During development of the zebrafish inner ear, regional patterning in the ventral half of the otic vesicle establishes zones of gene expression that correspond to neurogenic, sensory and non-neural cell fates. FGF and Retinoic acid (RA) signalling from surrounding tissues are known to have an early role in otic placode induction and otic axial patterning, but how external signalling cues are translated into intrinsic patterning during otic vesicle (OV) stages is not yet understood. FGF and RA signalling pathway members are expressed in and around the OV, suggesting important roles in later patterning or maintenance events. We have analysed the temporal requirement of FGF and RA signalling for otic development at stages after initial anteroposterior patterning has occurred. We show that high level FGF signalling acts to restrict sensory fates, whereas low levels favour sensory hair cell development; in addition, FGF is both required and sufficient to promote the expression of the non-neural marker *otx1b* in the OV. RA signalling has opposite roles: it promotes sensory fates, and restricts *otx1b* expression and the development of non-neural fates. This is surprisingly different from the earlier requirement for RA signalling in specification of non-neural fates via *tbx1* expression, and highlights the shift in regulation that takes place between otic placode and vesicle stages in zebrafish. Both FGF and RA signalling are required for the development of the otic neurogenic domain and the generation of otic neuroblasts. In addition, our results indicate that FGF and RA signalling act in a feedback loop in the anterior OV, crucial for pattern refinement.

## Author Summary

The vertebrate inner ear is a complex three-dimensional structure with hearing and balance functions. To form a functional ear in the embryo, it is crucial that the right cells develop at the right time and in the right place. These cells include the sensory hair cells that detect sound and movement, neurons that relay sensory information to the brain, and structural cells. We have investigated patterning and maintenance events in the developing ear of the zebrafish embryo. We show that two signalling pathways, FGF and Retinoic Acid (RA), act in an antagonistic manner to regulate the numbers of sensory hair cells that develop, together with the expression of a key gene, *otx1b*, required for the development of structural cells. However, the two signalling pathways act in concert to regulate the emergence of neuronal cells. Our data also indicate that FGF and RA signalling form a feedback loop, placing them at the heart of the regulatory network that ensures correct patterning is maintained in the ear. Both FGF and RA signalling are employed to generate hair cells and neurons for replacement therapies to treat hearing loss. Understanding the roles of FGF and RA signalling underpins the development of such therapies.

## Introduction

Most cell types of the inner ear arise from the otic placode, a region of specialised ectoderm lying adjacent to the developing hindbrain. To form the complex three-dimensional structure of the adult inner ear, cells in the otic region integrate information from both extrinsic and intrinsic factors over time, thereby gradually restricting the competence of the different regions in the emerging inner ear. In the developing zebrafish otic vesicle (OV), one of the first subdivisions to occur is the emergence of sensory, neurogenic and non-neural domains in the ventral otic epithelium. The sensory domain gives rise to the sensory patches or maculae at the anterior and posterior poles of the OV, consisting of sensory hair cells and supporting cells. Neurons of the statoacoustic ganglion (SAG) arise from the neurogenic domain in an anteroventral position. The non-neural domain, located ventrolaterally, gives rise to non-neural epithelium, including the ventral pillar of the lateral semicircular canal.

The emergence of these domains is a dynamic process, and key intrinsic regulators of these early otic cell fate decisions include *tbx1* and *otx1b*, which are both expressed in the otic placode and OV and code for transcription factors. The posteroventral OV expresses *tbx1*, a gene that has been associated with DiGeorge syndrome in humans. Both mice (*Tbx1* ^−/−^) and zebrafish (*van gogh* (*vgo*)) mutant for *tbx1* show an expansion of otic neurogenic anteroventral territories towards more posterior positions, demonstrating that Tbx1 functions to restrict neurogenesis in posterior otic regions [1], [2]. *otx1b* is expressed in a discrete ventrolateral domain in the zebrafish OV from around the 18 somite (18S) stage [3], [4], [5]. Injection of zebrafish embryos with a morpholino to *otx1b* results in loss of the non-neural epithelium separating the two maculae, together with the ventral semicircular canal pillar and lateral semicircular canal [6]. Expression of *otx1b* is lost in the ears of *vgo/tbx1* mutant embryos, suggesting that *otx1b* acts downstream of Tbx1 [7]. These observations, together with the dramatic ventral otic phenotypes observed when *tbx1* or *otx1b* are disrupted, led us to examine the regulatory signalling events that function upstream of *otx1b*.

In zebrafish, fibroblast growth factor (FGF) signalling emanating from rhombomere 4 at early somitogenesis stages is an early inducer of otic placodal fate [8], [9], [10], [11]. At late placode and early vesicle stages, FGF is required for the specification of anterior otic fates [12]. In addition, evidence from several model systems indicates that FGF signalling is required for the generation of otic sensory neuroblasts [8], [13], [14] (reviewed in [15]), which emerge from an anteroventral position in the zebrafish OV.

Retinoic acid (RA) signalling is another extrinsic factor that has been implicated in otic patterning at early developmental stages. RA plays a role in determining the extent of the region competent to respond to otic inducing signals [16]. Whilst FGF signalling is required for anteroventral otic fates, RA signalling has been implicated in the specification of posteroventral otic territories. The RA-producing enzyme gene *aldh1a2* is expressed in head mesenchyme posterior to the otic placode [17], [18], while *aldh1a3* is expressed in the developing ear from OV stages [19]. Recently, RA signalling from late gastrula stages has been shown to promote early otic non-neural fates. Here, it acts via *tbx1* to regulate the expression of the bHLH transcription factor *her9*, which is expressed in the posteroventral region of the zebrafish OV and is required for the repression of neurogenic fates [2].

**Figure 1:**
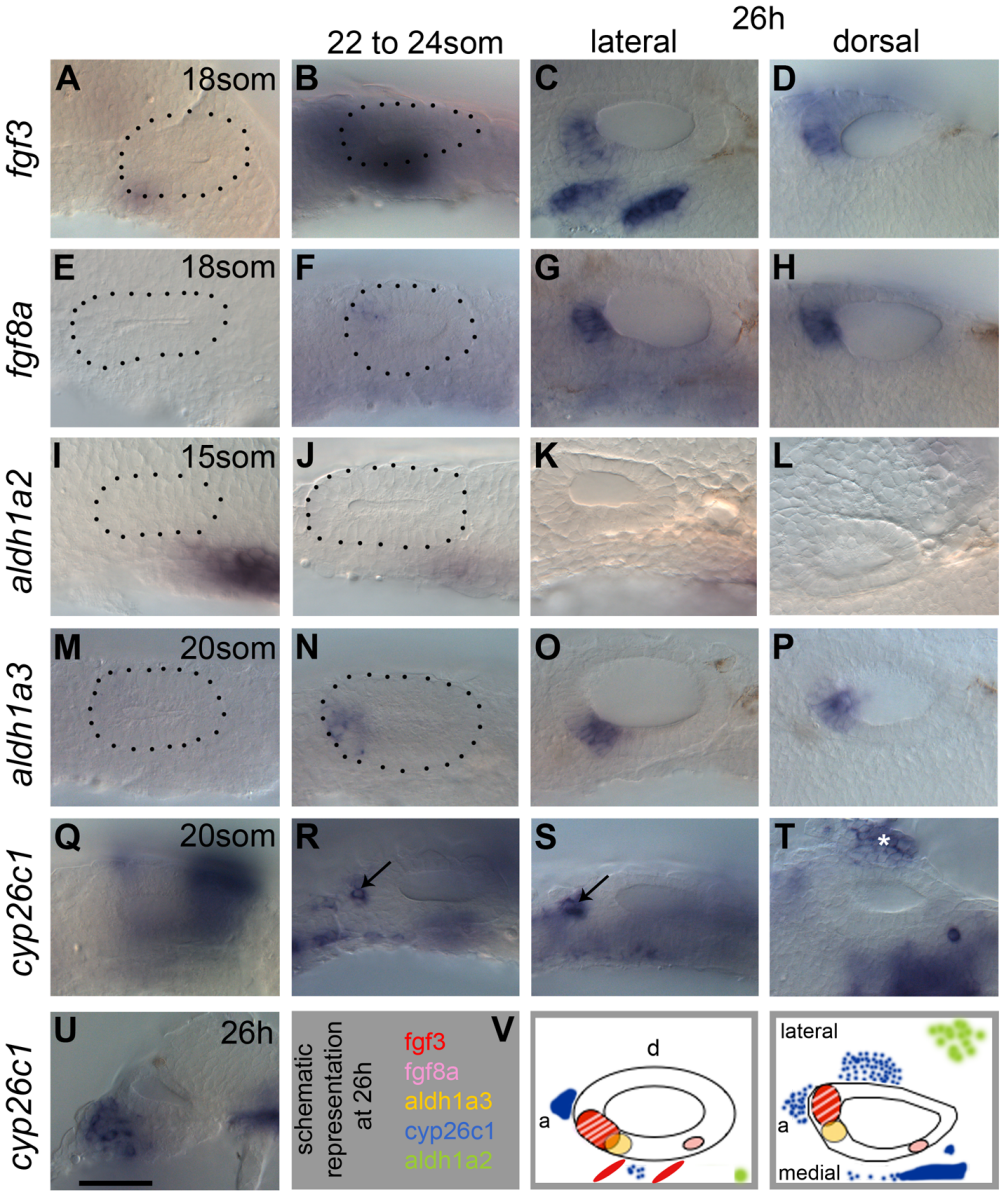
Expression of FGF and RA signalling pathway components during zebrafish OV development. All panels are lateral views with anterior to the left, apart from D,H,L,P,T (dorsal views; anterior left, lateral up), and U (transverse section, lateral left, dorsal up). (A–D) *fgf3* is expressed in pharyngeal pouch mesenchyme underlying the anteroventral OV from 18S. At 26 hpf (C,D,V), two stripes of expression underlie the OV in an anteroventral lateral position, and the OV itself expresses *fgf3* in the anterior. (E–H) *fgf8a* expression is very weak at early stages, but is strongly expressed in the anterior OV at 26 hpf (G,H,V). (I–L) At 15S, *aldh1a2* is strongly expressed in the mesenchyme posteroventral to the otic placode (I). At later stages, mesenchymal *aldh1a2* expression becomes weaker and shifts to a position further away from the OV (J–L,V). (M–P) Expression of *aldh1a3* can be detected in the OV from 22S in an anteroventral position. At 26 hpf, the *aldh1a3*-expressing cells are located in an anteroventral medial position in the OV (O,P,V). (Q–U) Expression of *cyp26c1* can be detected in periotic mesenchyme from early stages and at 26 hpf is expressed strongly in the hindbrain (out of focus). Groups of cells expressing *cyp26c1* abut the OV anteriorly (R,S, arrows) and ventrolaterally (T, asterisk). (V) Schematic representation of the expression domains of *fgf3*, *fgf8a*, *aldh1a3*, *cyp26c1* and *aldh1a2* in the OV at 26 hpf in a lateral and dorsal view. Scale bar: 50 µm.

Expression of FGF and RA pathway members persists at later OV stages (Fig. 1), both in surrounding tissues and in the OV epithelium, raising the intriguing possibility that these signalling pathways continue to act at later stages of inner ear development. Thus we investigated the roles of FGF and RA signalling after the initial induction and patterning phase to examine whether these two signalling pathways act during the refinement and maintenance phase of OV patterning (from the 18S stage onward). We have characterised the expression patterns of genes coding for components of both of these signalling pathways during later otic development. In addition, we have employed conditional approaches to manipulate FGF and RA signalling to analyse the late requirement of both FGF and RA signalling during OV stages and provide loss of function data for *aldh1a3*. We provide evidence that ventral otic *otx1b* expression, a marker of non-neural fates, requires ongoing high levels of FGF signalling, whereas lower levels of FGF favour sensory (hair cell) development. RA signalling, on the other hand, promotes sensory fates and restricts *otx1b* expression. In addition, we show that *tbx1* and *otx1b* expression are differentially regulated by RA signalling. This suggests that both FGF and RA signalling continue to function during late phases of ventral OV development. The analysis of *aldh1a3* morphant embryos pinpoints the anterior OV as the source of RA at these stages. Expression of *aldh1a3* in the OV is itself perturbed in embryos mutant for *fgf3* and *fgf8a*, or in embryos where FGF signalling has been blocked chemically, indicating that FGF signalling is required for *aldh1a3* expression at later stages. Moreover, we provide evidence that RA can suppress anterior otic *fgf3* and *fgf8a* expression. As *fgf3* and *fgf8a* start to be expressed in the OV before *otx1b* or *aldh1a3*, this suggests a temporal sequence of events that interconnects FGF and RA in a feedback loop.

## Results

### Expression of FGF and RA signalling pathway members in the zebrafish otic vesicle

To address the role of FGF and RA signalling in otic patterning at otic vesicle (OV) stages, we first examined the expression of pathway members in and around the OV from 18 hours post fertilisation/18 somites (18 hpf/18S). As previously reported, both *fgf3* and *fgf8a* are expressed in the anterior OV from around 18 hpf (Fig. 1A–H), with weak *fgf8a* expression in the posterior OV (Fig. 1G,H) [8]. In addition, strong expression of *fgf3* is observed in the pharyngeal pouches underneath the ventral OV floor, whereas *fgf8a* is expressed only weakly in this region (Fig. 1C,G). In addition, *fgfr1a*, *fgfr2* and *fgfr4*, but not *fgfr3*, are expressed in the OV in partially overlapping but distinct patterns (S1 Figure), indicating that the ventral OV is competent to respond to FGF signalling. *fgfr4* shows the strongest expression, in a ventromedial stripe at 22S. At 26 hpf, expression of *fgfr4* is excluded from the neurogenic region, but remains strongly expressed at the OV poles. *fgfr1a* and *fgfr2* are expressed in the posterior part of the OV weakly from 22S and expression persists at 26 hpf. Transverse sections through the OV at 26 hpf reveal that *fgfr1a* is expressed fairly ubiquitously, while *fgfr2* is expressed posteromedially (S1 Figure).

RA levels in the embryo are thought to reflect a balance between expression levels of RA-producing (*aldh*) and RA-degrading (*cyp*) enzyme genes. Two *aldh* genes are expressed in or near the zebrafish ear at the relevant stages. *aldh1a2* is expressed in the mesenchyme adjacent to the otic placode posteriorly at the 15S stage (Fig. 1I) [18]. As development proceeds, weak expression persists in the mesenchyme, but the distance of the expression domain from the OV increases (Fig. 1J–L), indicating that *aldh1a2* is not likely to influence later stages of OV development. This is further corroborated by the analysis of *aldh1a2/neckless* (*nls*) mutant embryos at 26 hpf, which show only subtle defects in OV patterning (S2 Figure). *aldh1a3* is expressed in the anteroventral OV from 20/21S onwards (Fig. 1M–P) [19]. At 20S, the RA-degrading enzyme gene *cyp26c1* is expressed in the hindbrain rhombomere 5/6 and in the head mesenchyme from the 6S stage (Fig. 1Q–U) [20]. As development proceeds, an additional expression domain is detected in a cluster of cells adjacent to the anterior of the OV (Fig. 1R,S). At 26 hpf, *cyp26c1* expression continues in rhombomere 5/6 of the hindbrain and in cells anterior and lateral to the OV, which are likely to form the main RA sink sites surrounding the OV (Fig. 1S–U). A schematic summary of these expression data at 26 hpf is shown in Fig. 1V. The four retinoic acid receptor genes show distinct and spatially regulated expression patterns in the zebrafish OV at 26 hpf (S1 Figure). Both *rarab* and *rargb* appear to mark the two developing sensory patches, while *rarga* shows a strikingly similar pattern to that of *tbx1*, with expression excluded from an anteroventral domain. *raraa* is only weakly expressed at the anterior of the OV. Taken together, these data raise the interesting possibility that FGF and RA signalling influence patterning or maintenance events in the OV between 18S and 26 hpf.

### Graded FGF signalling is required for otic vesicle patterning at later stages of development

We first tested whether FGF signalling is required during OV stages. To bypass the early requirements of FGF signalling in otic induction and anterior-posterior patterning, we exposed embryos to different concentrations of the chemical pan-FGF inhibitor SU5402 [21], [22], from 18/20S to 26 hpf. In our hands, SU5402 was a very effective inhibitor of the FGF response genes *etv4* and *dusp6* throughout the embryo, including in the OV (S3 Figure). To assess the effects of FGF inhibition on the OV at 26 hpf we used the following markers for different subregions of the OV: (1) To identify the non-neural domain, we used *tbx1*, which at 26 hpf is most strongly expressed in the posteroventral OV, and *otx1b*, which is expressed in the ventrolateral OV floor. (2) To identify the neurogenic domain, we analysed the expression of *neurog1* (expressed in the neurogenic region in the OV in specified neuronal cells and in some of the emerging neuroblasts) and *neurod1* (expressed in delaminating otic neuroblasts and those forming the statoacoustic ganglion beneath the OV). (3) To assess sensory patch spacing and development we analysed the expression of *sox2*, *atoh1a* and *tecta*, which are all expressed in the emerging utricular and saccular maculae (S4 Figure).

**Figure 2:**
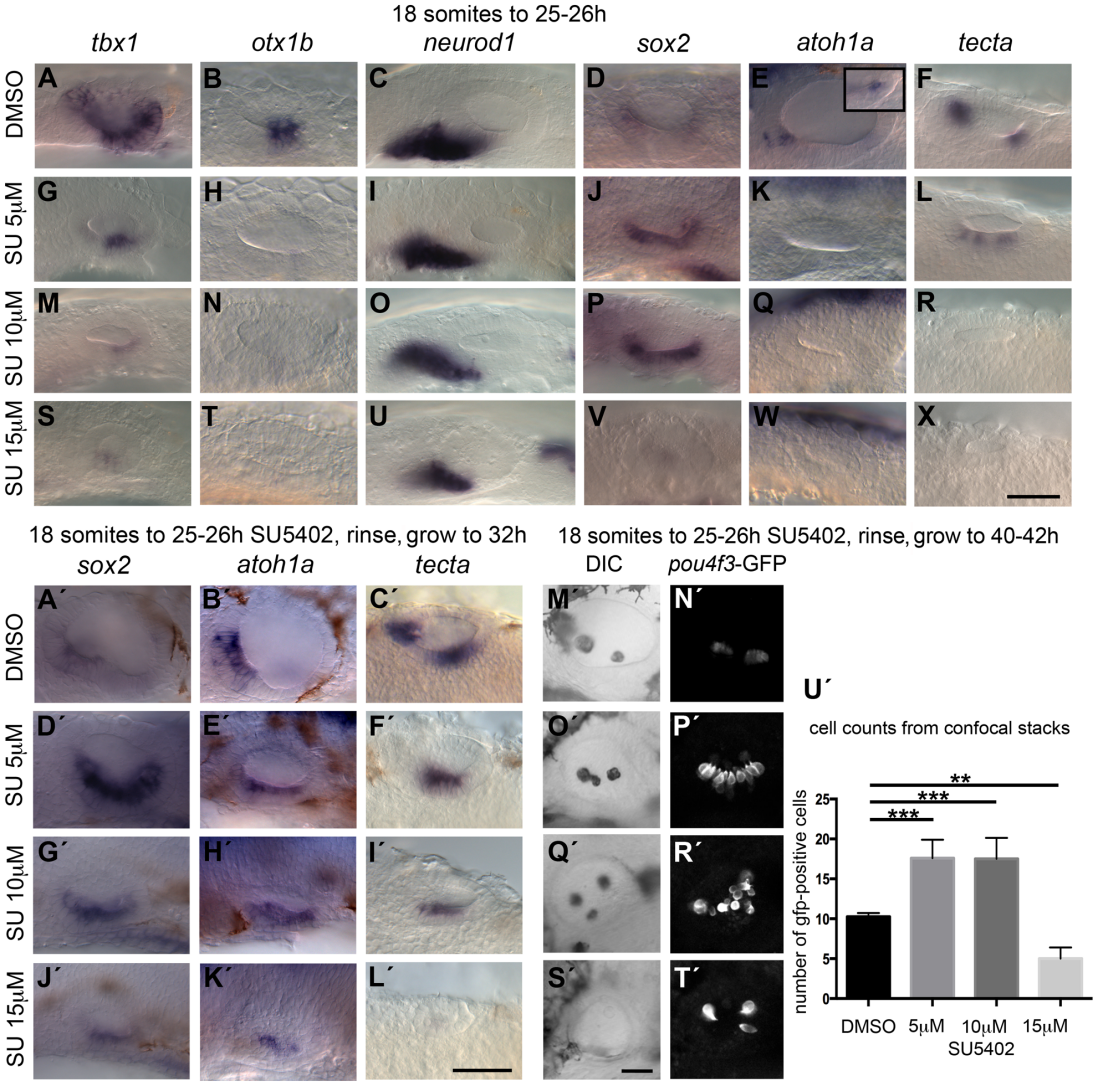
Inhibition of FGF signalling by SU5402 reveals a concentration-dependent effect of FGF signalling during OV development. Wild-type (WT) embryos were treated with DMSO or SU5402 from 18S to 26 hpf (A–X) or rinsed and grown on to 32 hpf (A′–L′) or 40–42 hpf (M′–T′). (A–F) WT embryos treated with DMSO show normal expression of non-neural markers *tbx1* (*n* = 51) and *otx1b* (*n* = 61; A,B), the neuronal marker *neurod1* (*n* = 64; C), and the sensory markers *sox2* (*n* = 56), *atoh1a* (*n* = 26) and *tecta* (*n* = 44; D–F). Boxed area in panel (E) depicts the *atoh1a* expression in posterior sensory patch in a different focal plane. (G–R) Treatment with 5 µM SU5402 (G–L) or 10 µM SU5402 (M–R) reduces the expression of *tbx1* (*n* = 56; G,I) and *neurod1* (*n* = 76; M,O). Expression of *otx1b* is lost (*n* = 32; H,N) and expression of *sox2* is extended in the ventral OV floor (*n* = 45; J,P). The expression of *atoh1a* is lost (*n* = 18; K,Q) and expression of *tecta* is weak but extended (*n* = 27; L, 5 µM SU5402) or lost (*n* = 40; R, 10 µM SU5402). (S–X) Treatment with 15 µM SU5402 reduces the expression of *tbx1* (*n* = 38; S) and *neurod1* (*n* = 65; U); expression of *otx1b* (*n* = 57), *sox2* (*n* = 58), *atoh1a* (*n* = 35), and *tecta* (*n* = 51) is lost (T,V–X). (A′–C′) WT embryos treated with DMSO show normal expression of the sensory markers *sox2* (*n* = 23), *atoh1a* (*n* = 31) and *tecta* (*n* = 25) at 32 hpf. (D′–I′) In embryos treated with 5 µM SU5402 (D′–F′) or 10 µM SU5402 (G′–I′), expression of *sox2* (*n* = 23) and *atoh1a* (*n* = 25) is extended in the ventral OV floor; *tecta* expression is misplaced in a single ventromedial patch (*n* = 24). (J′–L′) Treatment with 15 µM SU5402 reduces or abolishes the expression of *sox2* (*n* = 18), *atoh1a* (*n* = 16), and *tecta* (*n* = 14). (M′,N′) Tg(*pou4f3:GFP*) embryos treated with DMSO show normal hair cell patterns marked with GFP (*n* = 26). (O′,P′) In Tg(*pou4f3:GFP*) embryos treated with 5 µM SU5402, GFP-positive cells extended between the anterior and posterior sensory patch in the ventral OV floor (*n* = 18). (Q′,R′) In Tg(*pou4f3:GFP*) embryos treated with 10 µM SU5402, GFP-positive cells appear less orderly and are extended in the ventral OV floor (*n* = 12). (S′,T′) Treatment with 15 µM SU5402 reduced the number of GFP-positive cells in the OV (*n* = 14). (U′) Graphical representation of counts of GFP-positive cells at 40–42 hpf. Error bars represent standard error of the mean. One-way ANOVA with Dunnett's multiple comparison post-test: **p = 0.0065, ***p<0.0005. All panels are lateral views with anterior to the left, apart from the panels depicting *tecta* (dorsal views; anterior to the left, lateral up). Scale bar: 50 µm.

Embryos treated with 5, 10 or 15 µM SU5402 from the 18/20S stage onwards all showed a severe reduction (5, 10 µM) or loss (15 µM) of *tbx1* expression (Fig. 2G,M,S), and a loss of *otx1b* expression, as previously reported [8] (Fig. 2H,N,T), suggesting that FGF signalling is required for normal development of the non-neural domain. Concentrations of 20 µM SU5402 were toxic. Expression of *neurod1* decreased with rising SU5402 concentration (Fig. 2I,O,U), in line with results reported previously [14], confirming that FGFs are required for normal levels of otic neurogenesis (neuroblast specification and/or proliferation), even in the absence of *tbx1* expression. Interestingly, expression of sensory patch markers varied with the different inhibitor concentrations. Low and intermediate levels of SU5402 (5 and 10 µM) resulted in expansion of the *sox2* expression domain, which now extended across the entire ventral OV floor (Fig. 2J,P), whereas expression of *sox2* was lost at high SU5402 levels (15 µM; Fig. 2V). Expression of both *atoh1a* and *tecta*, more mature sensory patch markers, was decreased or lost at 26 hpf at all concentrations tested (Fig. 2).

To assess whether sensory development was blocked at the *sox2*-expressing stage during low and intermediate level SU5402 treatment, we treated wild-type (WT) and Tg(*pou4f3:gfp*) embryos, in which mature hair cells express GFP, with SU5402, washed at 26 hpf and grew on further to analyse *sox2*, *atoh1a* and *tecta* expression at 32 hpf in WT embryos (Fig. 2A′–L′) or GFP expression at 40–42 hpf in transgenic embryos (Fig. 2M′–U′). At these later stages, embryos exposed to 5 and 10 µM SU5402 now showed an up-regulation of *sox2* and *atoh1a* expression in the ventral OV floor, while *tecta* expression is misplaced (Fig. 2D′–I′), whereas all three markers were lost or reduced at high SU5402 levels (15 µM, Fig. 2J′–L′). The maculae in the Tg(*pou4f3:gfp*) transgenic embryos treated with 5 µM SU5402 appeared orderly but were enlarged and merged in the middle (Fig. 2D′–F′,P′), whereas at 10 µM SU5402 the merged maculae were more disarrayed (Fig. 2G′–I,′, R′). A significant increase in the number of GFP-positive cells, indicative of mature hair cells, was observed in these embryos (Fig. 2U′). This indicates that despite a block in hair cell differentiation during SU5402 treatment, the spatial domain of sensory development was expanded in embryos treated with low and intermediate concentrations of the FGF inhibitor, and that hair cell differentiation could proceed once FGF inhibition was relieved. There was a concomitant disruption to otolith formation, with ectopic and misplaced otoliths observed in the ears of treated embryos (Fig. 2O′,Q′,S′). At the highest SU5402 concentration, hair cells were almost entirely lost; the few that appeared might be early hair cells that formed before inhibitor treatment commenced. Interestingly, otoliths failed to form in embryos exposed to 15 µM SU5402, indicating that the otolith precursor-producing cells might be missing in these embryos as well (Fig. 2S′–U′).

Taken together, our results suggest a dose-dependent requirement for FGF signalling during OV development. High levels of FGF are required to maintain ventral, non-sensory and non-neural OV floor character as indicated by the loss or reduction of both *otx1b* and *tbx1* expression with low levels of SU5402. On the other hand, low levels of FGF signalling favour sensory development; a reduction in Fgf signalling is sufficient to lead to an up-regulation of sensory markers, and the differentiation of supernumerary hair cells, in the non-neural region of the OV. Our results also confirm a requirement for Fgf signalling for normal otic neurogenesis, as shown previously [8], [13], [14].

### FGF3 and FGF8A have different roles during otic vesicle development

**Figure 3:**
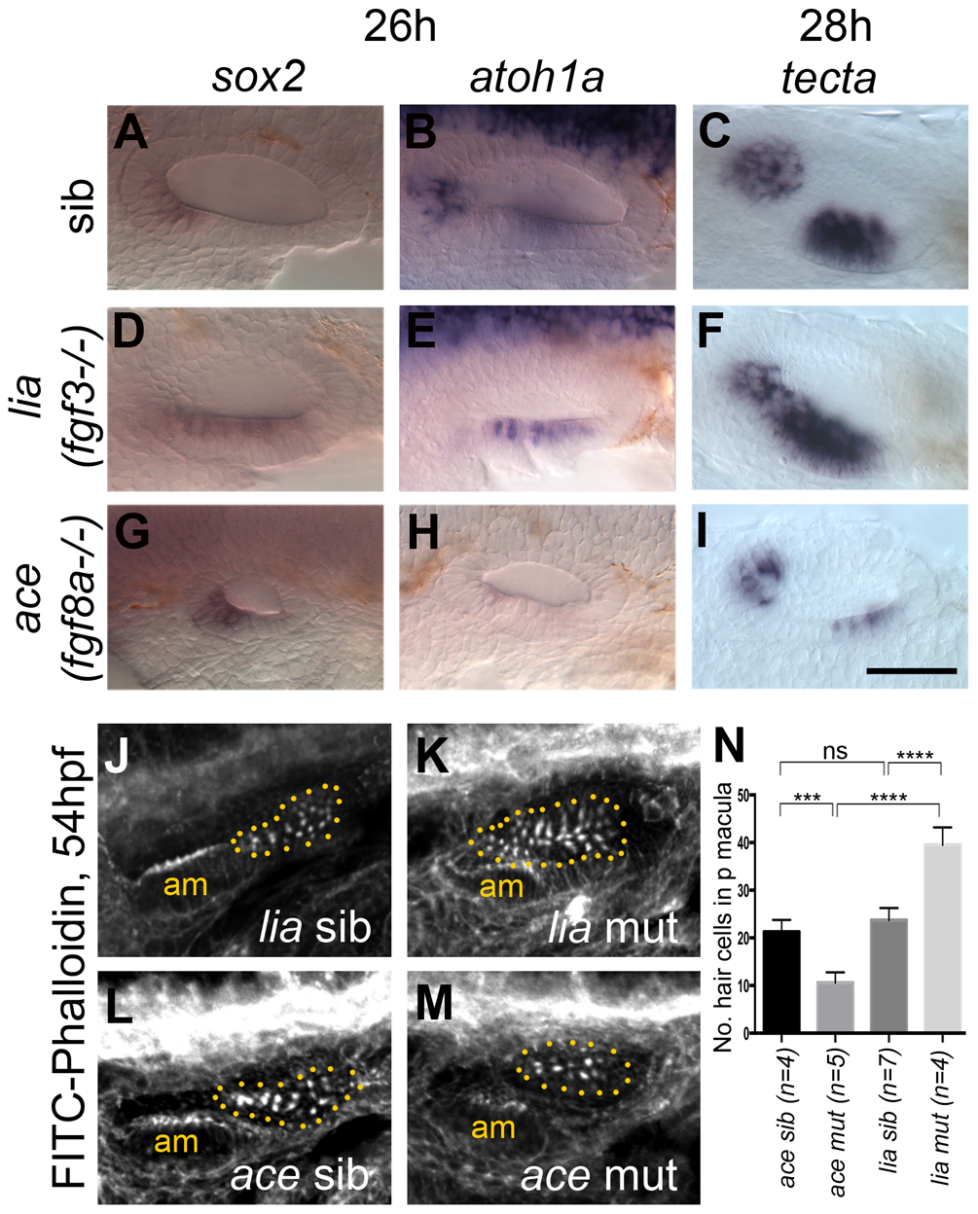
Expression of sensory markers in lia (fgf3^−/−^) and ace (fgf8a^−/−^) mutant embryos. (A–C) Phenotypically wild-type sibling (sib) embryos show normal expression of the sensory markers *sox2*, *atoh1a* and *tecta*. (D–F) In *lia (fgf3^−/−^)* embryos, expression of *sox2* (*n* = 12/41 embryos from a heterozygous cross), *atoh1a* (*n* = 13/45) and *tecta* (*n* = 14/42) is extended in the ventral OV floor. (G–I) In *ace (fgf8a^−/−^)* embryos, expression of *sox2* (*n* = 12/42), *atoh1a* (*n* = 10/39) and *tecta* (*n* = 14/54) shows normal spatial patterning, but while *sox2* expression levels are slightly increased, *atoh1a* and *tecta* levels are reduced. (J–N) Dotted yellow line demarcates the area of the posterior macula in which FITC-Phalloidin-positive hair bundles were counted. (J,L,N) Phenotypically wild-type sibling (sib) embryos show normal numbers of hair cells (*n* = 7 ears counted, *lia* sib; *n* = 4, *ace* sib). (K) In *lia (fgf3^−/−^)* embryos the posterior macula is enlarged; only FITC-phalloidin-positive cells that appeared posterior-like and were located in the demarcated area were counted (*n* = 4 mutant ears). (M) In *ace (fgf8a^−/−^)* embryos, hair cells show a normal spatial pattern, but the size of the maculae appears reduced (*n* = 6 mutant ears). (N) Graphical representation of counts of Phalloidin-positive cells at 53–56 hpf in the demarcated area. Error bars represent standard deviation. One-way ANOVA with Šídák's multiple comparison post-test: ***p<0.001, ****p<0.0001, ns = not significant. All panels are lateral views with anterior to the left, apart from the panels depicting *tecta*, which are dorsal views with anterior to the left, lateral up. Scale bar: 50 µm.

We were intrigued by the strong requirement for FGF signalling in the ventral OV floor revealed by the SU5402 treatments. Given the differences in expression patterns between *fgf3* and *fgf8a* in the pharyngeal pouch region underneath the OV (Fig. 1), and in their receptor specificity [23], we tested whether the two genes had different roles in OV patterning. To distinguish FGF3 from FGF8A signalling we employed *lia (fgf3^−/−^*) and *ace (fgf8a^−/−^)* mutant embryos. According to the literature, the *fgf8a^ti282a^* allele ‘strongly or completely’ inactivates the *fgf8a* gene [24], but the severity of the OV phenotype can vary in these mutants [8]. The observed effects described below could be detected across the range of OV size reduction that occurs in *ace (fgf8a^−/−^)* mutants. The *fgf3^t21142^* allele is likely to be null [25]. We examined expression of a subset of markers used in the SU5402 treatments at 26–28 hpf. Interestingly, with regard to sensory markers, *lia (fgf3^−/−^)* and *ace (fgf8a^−/−^)* mutant embryos showed qualitatively distinct phenotypes: in embryos deficient for *fgf3*, the two maculae appeared closer together or merged (Fig. 3D–F; see also [12]), resembling the phenotype observed at low levels of SU5402 treatment, whereas in *fgf8a*-deficient embryos, two sensory patches developed with the correct spacing in the smaller ear, but were reduced in size (Fig. 3G–I) (see also [8]). This was corroborated in *lia (fgf3^−/−^)* and *ace (fgf8a^−/−^)* mutant embryos stained for actin and analysed at 54 hpf. In *lia (fgf3^−/−^)* embryos the anterior and posterior maculae remained juxtaposed, and the posterior macula contained more hair cells than normal (Fig. 3J,K,N), whereas in *fgf8a*-deficient *ace* embryos the two sensory patches developed with the correct spacing in the smaller ear, but were reduced in size, with fewer hair cells in the posterior patch (Fig. 3L–N). We never observed a fusion of the sensory patches either at 26–28 hpf or at 54 hpf as has been reported for 1 out of 8 *ace (fgf8a^−/−^)* mutant embryos analysed at 5 dpf [8]. Expression of *tbx1* and *otx1b* was also affected differently in each of the FGF mutants at 26 hpf. In *lia (fgf3^−/−^)* mutants, *tbx1* expression extended ectopically into the anteroventral domain (normally devoid of *tbx1* expression; S5 Figure), most likely due to earlier functions of *fgf3* during otic development. In addition, *otx1b* expression was reduced (S5 Figure), although appeared normal at later stages [12]. By contrast, both genes were patterned normally, albeit in smaller domains, in the smaller *ace (fgf8a^−/−^)* mutant ear (S5 Figure) [8].

**Figure 4:**
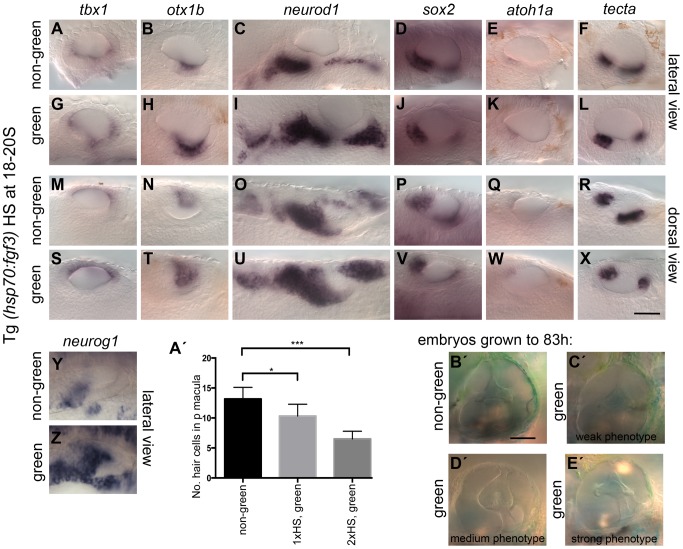
Effects of fgf3 over-expression from the 18 somite stage on otic vesicle patterning. Tg(*hsp70:fgf3*) embryos were heat shocked (HS) from 18S and sorted as non-green, non-transgenic siblings (A–F, M–R, Y, A′) or green transgenic embryos (G–L, S–X, Z, B′). (A–F, M–R, A′) In non-green embryos, expression of the non-neural markers *tbx1* (*n* = 79; A,M) and *otx1b* (*n* = 52; B,N), the neuronal marker *neurod1* (*n* = 18; C,O), and the sensory markers *sox2* (*n* = 11), *atoh1a* (*n* = 40), *tecta* (*n* = 32) (D–F, P–R) and the neurogenic marker *neurog1* (Y, *n* = 13) is normal. (G–L, S–X, B′) In Tg(*hsp70:fgf3*) embryos sorted as green, the expression of *tbx1* is unaffected or slightly up-regulated (*n* = 97; G,S), while expression of *otx1b* (*n* = 56; H,T), *neurog1* (*n* = 25; Z) and otic and non-otic *neurod1* (*n* = 35; I,U) is strongly up-regulated. The expression of *sox2* (*n* = 24), *atoh1a* (*n* = 83) and *tecta* (*n* = 100) is slightly reduced and the anterior part of the posterior domain marked by *tecta* is missing (J–L, V–X). (A′) Graphical representation of counts of GFP-positive sensory hair cells (*pou4f3:GFP* expression) in the demarcated area from embryos heterozygous for both Tg(*hsp70:fgf3*) and Tg(*pou4f3:GFP*). Error bars represent standard deviation. One-way ANOVA with Šídák's multiple comparison post-test: *p<0.05, ***p<0.001. (B′–E′) Live images of Tg(*hsp70:fgf3*) embryos HS at 18S and grown to 83 hpf. The inner ear morphology of non-green non-transgenic embryos appears normal (*n* = 10; B′). (C′–E′) Green Tg(*hsp70:fgf3*) embryos display only minor ventral patterning defects; the ventral pillar for the lateral semicircular canal is always present (*n* = 10; C′–E′), but is sometimes displaced posteriorly (E′). Scale bar: 50 µm.

The role FGF3 plays in otic patterning is further strengthened by over-expression of FGF3 using the Tg(*hsp70:fgf3*)-line (Fig. 4). The space between the two sensory patches was increased due to a specific reduction of the posterior sensory patch and instead, non-neurogenic (*otx1b*) and neuronal (*neurog1, neurod1*) markers were up-regulated (Fig. 4A–Z). Hair cell numbers in the posterior macula were specifically reduced at 49–53 hpf (Fig. 4A′), but overall ear morphology was only mildly perturbed (Fig. 4B′–E′). These data suggest that FGF3 is mainly responsible for the correct spacing of the sensory patches in the ventral otic floor, and influences hair cell number in the posterior macula. We speculate that the underlying pharyngeal pouches act as a source for FGF3 in this process, but further experiments are needed to test this conclusively. The smaller ear and reduced sensory patches in *ace* mutants are likely to reflect the earlier functions of FGF8A in otic induction, together with its requirement for sensory and neuronal development, as previously reported [26], [27].

### RA is required for the spatial patterning of the ventral otic vesicle

**Figure 5:**
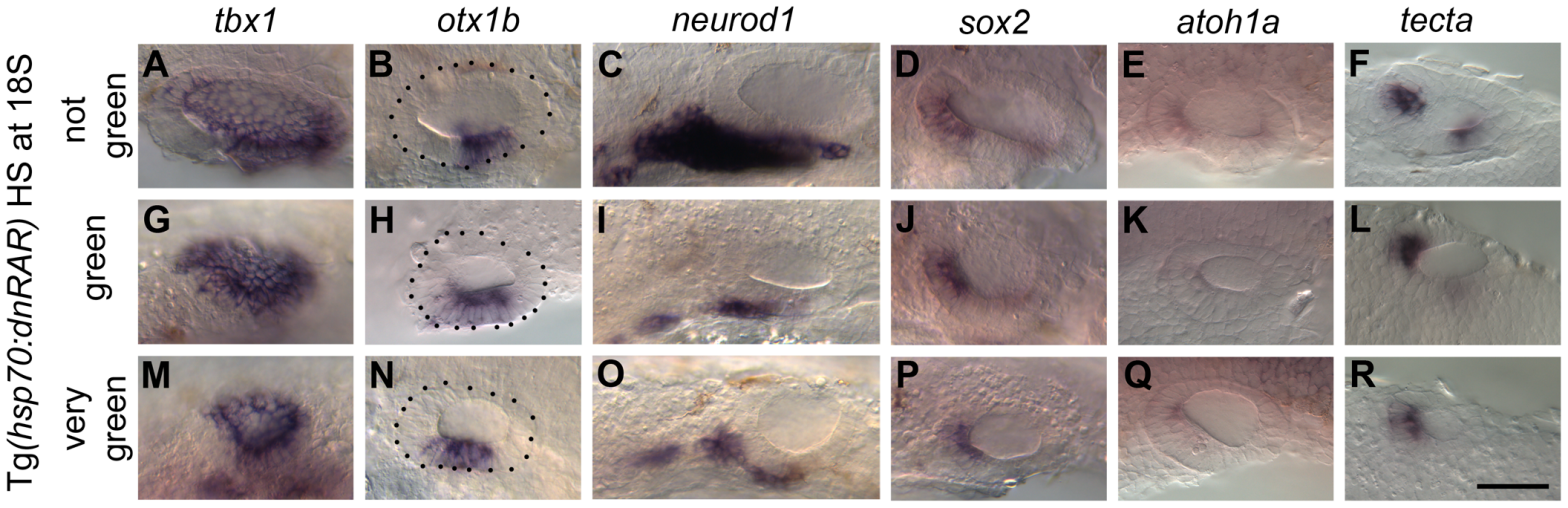
Heatshock of *Tg(hsp70:dnRAR)* embryos leads to an expansion of otic *otx1b* and a loss of *neurod1* expression. Tg*(hsp70:dnRAR*) embryos were heat shocked at 18S and sorted into non-green (non-transgenic siblings), green and very green embryos. (A–F) Non-transgenic sibling embryos show normal pattering of the OV assessed by the exp`ression of the non-neural markers *tbx1* and *otx1b* (A,B), the neuronal marker *neurod1* (C), and the sensory markers *sox2*, *atoh1a* and *tecta* (D–F) (at least 50 embryos per marker). (G–R) Embryos with low (G–L) or very low (M–R) levels of RA signalling show only a slight change in *tbx1* expression (*n* = 46; G,M), but expression of *otx1b* is expanded anteroventrally (*n* = 109; H,N) and expression of *neurod1* is reduced (*n* = 60; I,O). Expression of *sox2* (*n* = 106), *atoh1a* (*n* = 150) and *tecta* (*n* = 98) is reduced posteriorly (J–L,P–R) and anteriorly (P–R). All panels are lateral views with anterior to the left, apart from the panels depicting *tecta* (dorsal views; anterior to the left, lateral up). Scale bar: 50 µm.

RA signalling has been shown to regulate otic *tbx1* expression positively at early (late gastrula, 10 hpf) stages in zebrafish [2], a result that we were able to replicate with DEAB (RA inhibitor) treatment, albeit at higher concentrations (S6 Figure). To investigate the role of RA signalling during ventral OV development at later stages, we used a number of different approaches. Firstly, we used the Tg(*hsp70:dnRAR*) line to block RA signalling in a conditional manner, by heat-shocking embryos from heterozygous incrosses from 18S/20S (Fig. 5; S7 Figure). To our surprise, at these later stages of OV development, *tbx1* expression was no longer dependent on RA signalling and persisted in the OV, even in embryos expressing high levels of GFP in the OV, indicative of strong repression of RA signalling. There even appeared to be some ectopic expression of *tbx1* in the anteroventral domain (Fig. 5G,M). In all cases, *otx1b* expression was expanded anteriorly (Fig. 5H,N), while *neurod1* expression was severely reduced (Fig. 5I,O). The expansion of the *otx1b* domain occurred despite the overall reduction in size of the otic vesicle as a result of the *dnRAR* over-expression. Treatment with the chemical RA-inhibitor DEAB revealed that in addition to *neurod1*, the expression of the two neuronal markers *neurog1* (expressed in specified neuroblasts in the OV and some delaminated neuroblasts) and *isl1* (initiated after cell cycle exit in delaminated neuroblasts) is reduced in embryos with blocked RA signalling (S8 Figure). The reduction observed in *neurod1*-positive cells in embryos in which RA signalling had been abolished from 18S was more pronounced than after FGF inhibition, suggesting that the neuroblast lineage requires RA as well as FGF for proper development. In addition, sensory development was perturbed in embryos with reduced or blocked RA signalling (Fig. 5D–F, J–L, P–R).

**Figure 6:**
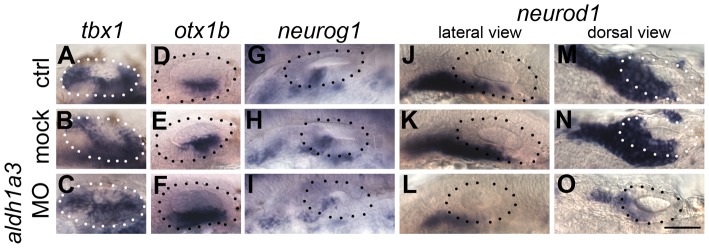
*aldh1a3* is required to pattern the zebrafish otic vesicle. (A–C) Control uninjected (A, *n* = 36) and mock-injected (B, *n* = 14) embryos show normal expression of *tbx1*. Embryos injected with the *aldh1a3* morpholino (C) show an expansion of expression of *tbx1* in the anteroventral part of the OV (*n* = 38/46). (D–F) Control uninjected (D, *n* = 98) and mock-injected (E, *n* = 25) embryos show normal expression of *otx1b*. Embryos injected with the *aldh1a3* morpholino (F) show a marked expansion of *otx1b* expression in the anteroventral part of the OV (*n* = 95/122). (G–I) Control uninjected (G, *n* = 11) and mock-injected (H, *n* = 9) embryos show normal expression of *neurog1*. Embryos injected with the *aldh1a3* morpholino (I) show a marked reduction of *neurog1* expression in the OV (*n* = 33). (J–O) Control uninjected (J,M, *n* = 42) and mock-injected (K,N, *n* = 18) embryos show normal expression of *neurod1*. Embryos injected with the *aldh1a3* morpholino (L,O) show a marked reduction of *neurod1* expression (*n* = 70/76). (A–L) Lateral views; (M–O) dorsal views; anterior to the left. Scale bar: 50 µm.

The expression of *aldh1a3* in the anterior otic vesicle and the lack of otic phenotype in *aldh1a2 (nls)* mutants suggested that *aldh1a3* is the likely source of RA signalling to pattern the otic epithelium at OV stages. To test this, we injected a translation-blocking morpholino to knock down *aldh1a3* function from early cleavage stages. The results corroborated the dnRAR data presented above, but were even more dramatic: in *aldh1a3* morphants, the *otx1b* expression domain expanded to cover the entire otic vesicle floor, while *tbx1* expression was slightly expanded into the anteroventral domain. Otic *neurog1* and *neurod1* expression was severely down-regulated (Fig. 6). Importantly, the morpholino did not result in any significant change in overall otic vesicle size (Fig. 6D–F). Taken together, these data indicate that *aldh1a3* is the source for RA signalling in the OV, where it is required to restrict *otx1b* expression in anterior OV regions and to establish or maintain normal levels of otic *neurog1* and *neurod1* expression.

**Figure 7:**
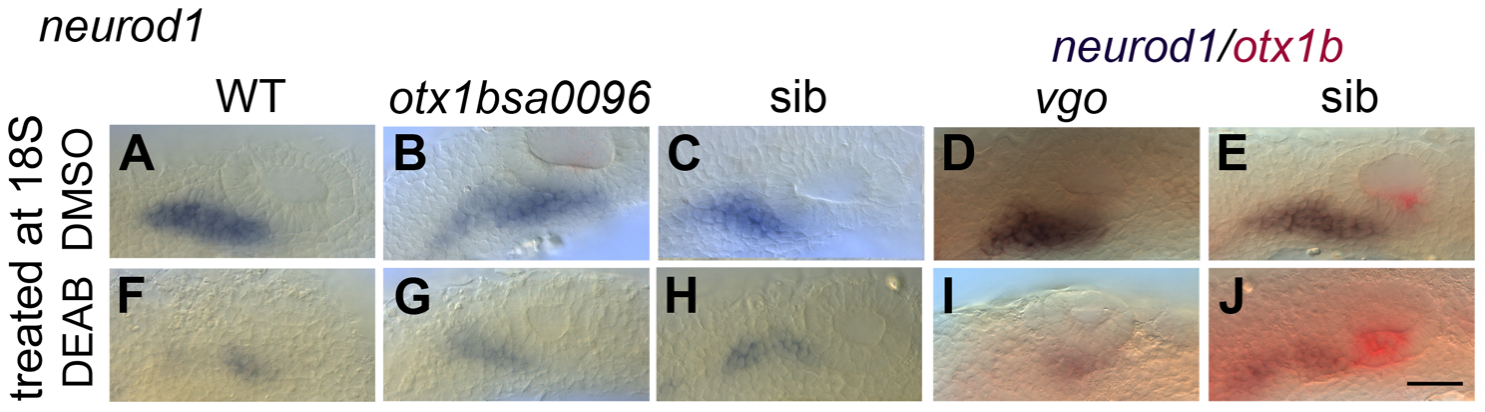
RA signalling is required for normal levels of otic *neurod1* expression in the absence of *tbx1* and *otx1b* activity. Embryos were treated with DMSO or DEAB from 18S to 26 hpf. (A) WT embryos treated with DMSO show normal expression of *neurod1* (*n* = 10). (F) Treatment of WT embryos with 400 µM DEAB (*n* = 18) leads to a reduction in the size of the OV and a reduction *neurod1* expression. (B) In *otx1b^sa96−/−^* embryos, expression of *neurod1* is shifted posteriorly (*n* = 4/16 embryos from a heterozygous cross). (G) In *otx1b^sa96−/−^* embryos treated with DEAB, the OV is smaller and expression of *neurod1* is reduced (*n* = 9/37). (C) In *otx1b^sa96^* sibling (sib; wild-type or heterozygote) embryos, expression of *neurod1* is normal (*n* = 12/16 embryos from a heterozygous cross), but levels are reduced after treatment with DEAB (*n* = 28/37; H). (D) In *tbx1^−/−^* (*vgo*) embryos, expression of *otx1b* is lost and expression of *neurod1* is shifted posteriorly (*n* = 9/38). (I) *tbx1^−/−^* embryos treated with DEAB (*n* = 16/62) do not express *otx1b* (lack of red stain); expression of *neurod1* is still shifted posteriorly, but is reduced. (E) *vgo* sibling embryos treated with DMSO show normal expression of *neurod1* (purple) and *otx1b* (red) (*n* = 29/38 embryos from a heterozygous cross). (J) Treatment of sibling embryos with DEAB (*n* = 46/62) leads to a reduction in *neurod1* expression. All panels are lateral views with anterior to the left. Dotted outline marks position of the OV; vertical lines mark posterior extent of the *neurod1*-expressing domain relative to the OV. Scale bar: 50 µm.

To test whether RA functions directly in the neuroblast lineage or acts indirectly by suppressing *otx1b* expression in the neurogenic domain, we employed *otx1b^−/−^* and *vgo* (*tbx1^−/−^*) mutant embryos (Fig. 7). *vgo* mutant embryos lack expression of *otx1b* in the OV, and in both *otx1b^−/−^* and *vgo* (*tbx1^−/−^*) embryos the otic neurogenic domain is increased and neuroblasts emerge in a more posterior position in the OV floor (Fig. 7B,D) [2], [28]. We reasoned that inhibiting RA signalling in the absence of *otx1b* expression should reveal *otx1b*-independent regulatory roles of RA signalling on neuroblast development. We cultured WT, *otx1b^−/−^*, *vgo* (*tbx1^−/−^*) mutant and sibling embryos together in the RA inhibitor DEAB (400 µM) from 18S to 26 hpf and analysed expression of *neurod1*. While expression of *neurod1* is normal in embryos treated with DMSO (Fig. 7A–E) it occurred in a smaller domain at reduced levels in WT, sibling and mutant embryos treated with DEAB (Fig. 7F–J), suggesting that RA is required directly in the neuroblast lineage, rather than acting through suppression of *otx1b* function.

**Figure 8:**
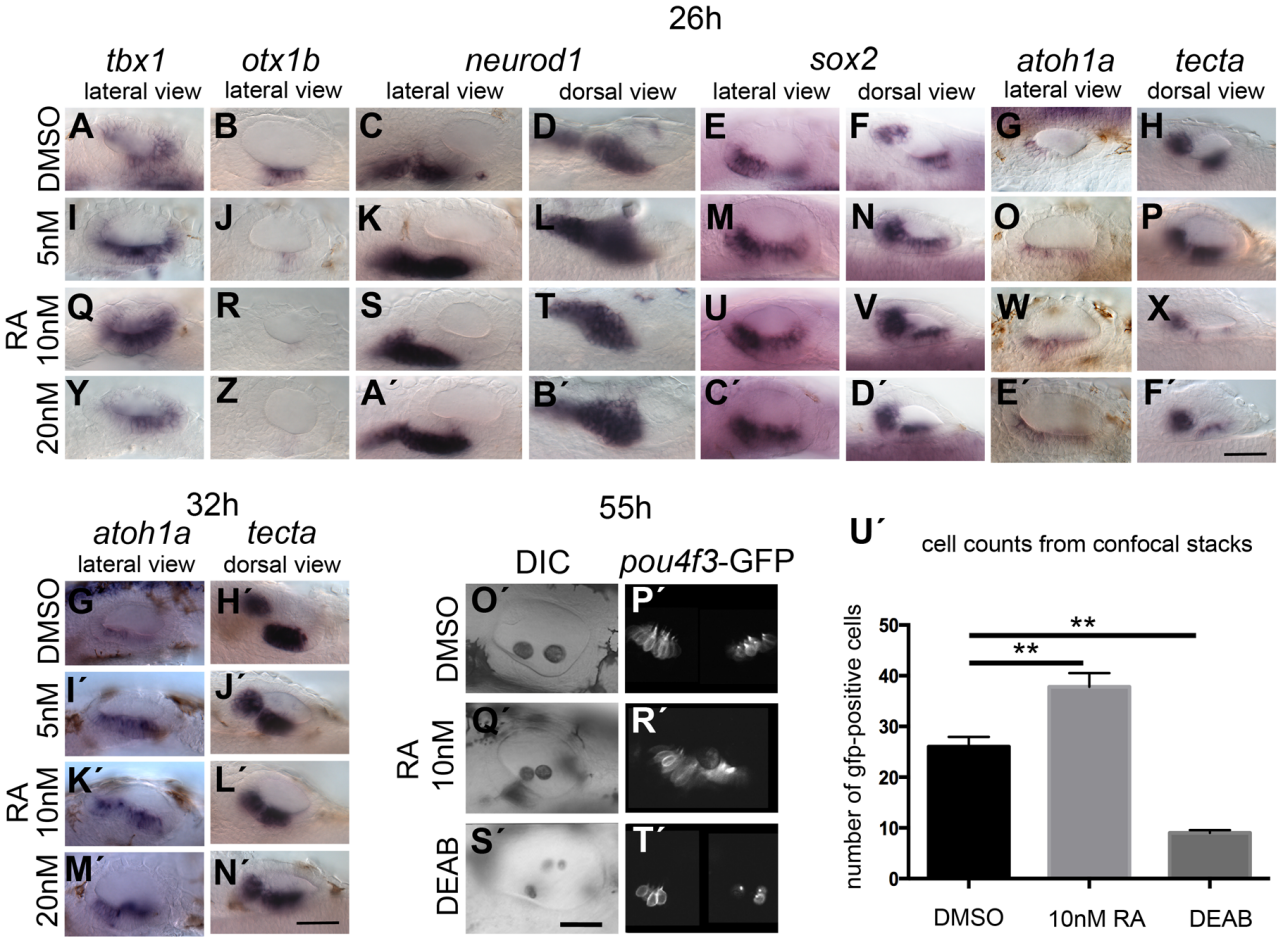
Effects of RA treatment on otic vesicle patterning. WT embryos were treated with DMSO or RA from 20S to 26 hpf (A–F′), 32 hpf (G′–N′) or 55 hpf (O′–T′). (A–H) WT embryos treated with DMSO show normal expression of non-neural markers *tbx1* (*n* = 71; A) and *otx1b* (*n* = 52; B), the neuronal marker *neurod1* (*n* = 50; C,D), and the sensory markers *sox2* (*n* = 54), *atoh1a* (*n* = 63) and *tecta* (*n* = 40) (E–H). (I–X) Treatment with 5 nM RA (I–P) or 10 nM RA (Q–X) results in expanded *tbx1* expression (*n* = 80/110; I,Q), down-regulation of *otx1b* expression (*n* = 104; J,R), and expansion of the *neurod1* expression domain (*n* = 94/100). The expression of *sox2* (*n* = 72/86) and *atoh1a* (*n* = 68) is extended in the ventral OV floor (M–O, U–W). Expression of *tecta* (*n* = 109) is reduced, but *tecta*-positive cells emerge in the inter-patch space (P,X). (Y–F′) Treatment with 20 nM RA results in a slight down-regulation, but anteroventral expansion, of *tbx1* expression (*n* = 42/62; Y), down-regulation of *otx1b* expression (*n* = 64; Z), and an expansion of the *neurod1* expression domain (*n* = 55; A′,B′). Expression of *sox2* extends in the ventral OV floor (*n* = 51/62; C′,D′), while the expression of *atoh1a* (*n* = 38/40) is extended. Expression of *tecta* (*n* = 44/54) is down-regulated, but *tecta*-positive cells emerge in the inter-patch space (E′,F). (G′,H′) WT embryos treated with DMSO show normal expression of the sensory markers *atoh1a* (*n* = 34) and *tecta* (*n* = 49) when grown to 32 hpf. (I′–L′) In embryos treated with 5 nM (I′,J′), 10 nM (K′,L′) or 20 nM RA (M′–N′), expression of *atoh1a* extends in the ventral OV floor (*n* = 65, all concentrations). The expression of *tecta* (*n* = 96, all concentrations) indicates that the two sensory patches are closer together or fused (J′,L′,N′). (O′–U′) Tg(*pou4f3:GFP*) embryos treated with DMSO show normal hair cell development in two clearly separated sensory patches (*n* = 23; O′,P′). In Tg(*pou4f3:GFP*) embryos treated with 10 nM RA, GFP-positive cells differentiate between the anterior and posterior sensory patch in the ventral OV floor (*n* = 18; Q′,R′). In Tg(*pou4f3:GFP*) embryos treated with DEAB, GFP-positive cells are severely reduced in the OV (*n* = 13; S′,T′). (U′) Graphical representation of the number of GFP-positive cells per ear for each treatment. Error bars represent standard error of the mean. One-way ANOVA with Dunnett's multiple comparison post-test: RA vs. DMSO: **p = 0.0082; DEAB vs. DMSO: **p = 0.0019. All panels are lateral views with anterior to the left, apart from the panels depicting *tecta* (dorsal views; anterior to the left). Scale bar: 50 µm.

Next we addressed the effects of exogenous RA signalling on OV development. We cultured embryos in 5, 10 and 20 nM RA at the 18S/20S stages, and assessed non-neural, neurogenic and sensory markers at 26 hpf (Fig. 8). Exogenous RA signalling affected the non-neural markers *tbx1* and *otx1b* differently. While an up-regulation of *tbx1* expression was observed, with expression extending anteriorly (Fig. 8I,Q,Y), otic *otx1b* expression was severely down-regulated or lost in embryos treated with 5,10 or 20 nM RA (Fig. 8J,R,Z). In addition, the size of the otic *neurod1*-expressing domain was expanded in embryos treated with the lower concentration of RA (Fig. 8K,L) but normal or only a little expanded in embryos treated with 10 or 20 nM RA (Fig. 8K,L,S,T,A′,B′). Analysis of the neuronal markers *neurog1* and *isl1* in concert with *neurod1* reveals that while delaminating (*neurod1*-positive) and postmitotic (*isl1*-positive) neuroblast domains are increased in embryos treated with RA, the *neurog1*-positive domain in the OV is unchanged or slightly decreased in embryos treated with RA (S8 Figure). This suggests that RA promotes the transition from the *neurog1*-expressing to the *neurod1*-expressing state during neuroblast maturation. Expression of *sox2* was expanded, and as with the low level SU5402 treatments, expression of *tecta* was blocked (Fig. 8). The effect on sensory markers was also seen if treatment continued for longer time points, up to 32 hpf. Supernumerary *atoh1a*-positive cells emerged in the maculae and in the OV floor (Fig. 8I,K′M′), and the spacing between the two sensory patches was reduced (*tecta*, Fig. 8J′,L′,N′). An increase in the number of GFP-positive cells at 55 hpf was also observed in Tg(*pou4f3:gfp*) embryos treated with 10 nM RA from 20S (Fig. 8Q′,R′,U′), whereas inhibition of RA signalling by DEAB arrested hair cell and otolith development (Fig. 8S′,T′,U′).

Taken together, our results support a model where RA, most likely emanating from the *aldh1a3*-expressing cells in the anterior OV, is required and sufficient to repress *otx1b* expression in the emerging sensory and neurogenic domains of the anterior OV. In addition, development of otic neuroblasts seems to depend critically on RA signalling: inhibition of RA signalling leads to a decrease in *neurog1* and *neurod1* expression (most likely indicating a smaller neuronal domain with fewer emerging neuroblasts), suggesting that levels of RA have to be tightly regulated for proper neurogenesis to occur. Most intriguingly, elevated levels of RA signalling are sufficient to induce ectopic sensory hair cell development in the ventral OV, a phenotype that mimics the low level inhibition of FGF signalling, suggesting that RA and FGF signalling have opposing roles in otic sensory development.

### FGF and RA signalling in the developing ear—A possible feedback loop

**Figure 9:**
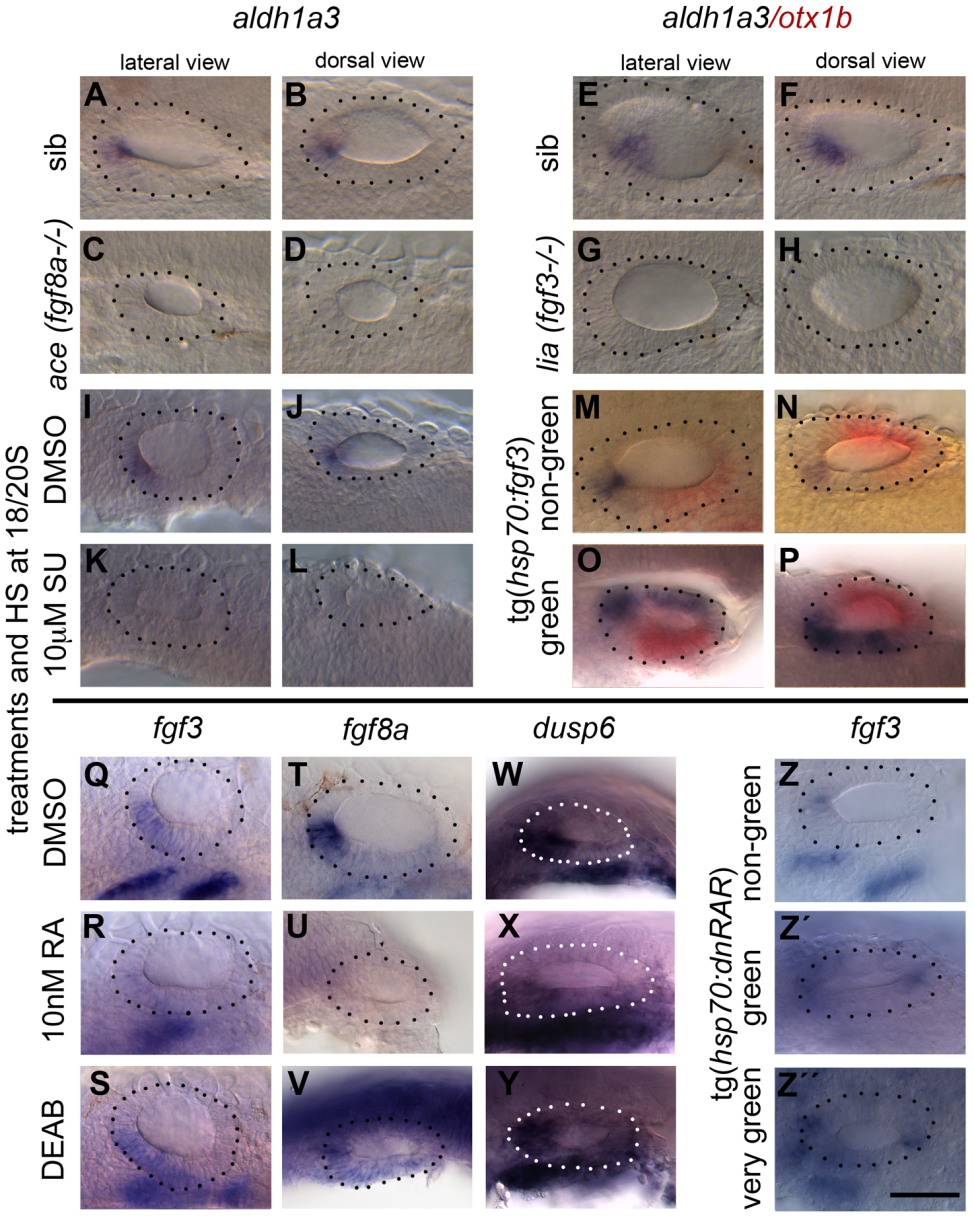
RA signalling and FGF signalling are linked in a regulatory feedback loop in the ear. Embryos were treated with DMSO, SU5402 and DEAB from 18S to 26 hpf, with RA from 20S to 26 hpf, and heatshock was performed at the 18/20S stage. (A–H) *aldh1a3* expression is normal in sibling embryos (*n* = 24/31 embryos from a heterozygous cross (*ace* sib), *n* = 31/39 (*lia* sib)) but lost in embryos mutant for *fgf8a* (*ace*, *n* = 7/31; C,D) or *fgf3* (*lia*, *n* = 8/39; G,H). (I–L) *aldh1a3* expression is normal in WT embryos treated with DMSO (*n* = 15; I,J), but lost in embryos treated with 10 µM SU5402 (*n* = 35; K,L). (M–P) Tg(*hsp70:fgf3*) embryos were heat shocked (HS) from 18S and sorted as non-green non-transgenic siblings (M,N), or green transgenic embryos (O,P), and double stained for *aldh1a3* (blue) and *otx1b* (red). In non-green non-transgenic embryos, expression of *aldh1a3* is normal (*n* = 31). In green Tg(*hsp70:fgf3*) embryos, expression of *aldh1a3* and *otx1b* is up-regulated (*n* = 48). (Q–S) *fgf3* expression is normal in WT embryos treated with DMSO (*n* = 15; Q) but down-regulated in the otic epithelium in embryos treated with 10 nM RA (*n* = 28; R); the domain is expanded in embryos treated with DEAB (*n* = 23; S). (T–V) *fgf8a* expression is normal in WT embryos treated with DMSO (*n* = 16; T) but down-regulated in the otic epithelium in embryos treated with 10 nM RA (*n* = 22; U) and expanded posteroventrally in embryos treated with DEAB (*n* = 19; V). (W–Y) *dusp6* expression is normal in WT embryos treated with DMSO (*n* = 9; W) but down-regulated in the otic epithelium in embryos treated with 10 nM RA (*n* = 26; X) and expanded posteroventrally in embryos treated with DEAB (*n* = 17; Y). (Z–Z″) Tg(*hsp70:dnRAR*) embryos were heat shocked (HS) from 18S and sorted as non-green non-transgenic siblings (Z) or green transgenic embryos (Z′,Z″). In non-green, non-transgenic embryos, the expression of *fgf3* is normal (*n* = 18; Z). In green and very green Tg(*hsp70:dnRAR*) embryos the expression *fgf3* is up-regulated (*n* = 28; Z′,Z″). Dotted line demarcates the OV. Scale bar: 50 µm.

The similarities between the effect of FGF inhibition and RA over-exposure with regard to sensory markers prompted us to explore whether FGF and RA signalling influence each other in the OV. Since *fgf*3 and *fgf8a* are expressed in the otic epithelium before *aldh1a3* expression can be observed, we asked whether FGF signalling is required for *aldh1a3* expression and sufficient to induce it ectopically. Otic *aldh1a3* expression was lost in *ace (fgf8a^−/−^)* and *lia (fgf3^−/−^)* mutant embryos, and in embryos treated with 10 µM SU5402 at 18–20S (Fig. 9A–L), suggesting that high levels of FGF signalling are required for otic *aldh1a3* expression. In addition, elevating FGF3 levels by heat-shocking Tg(*hsp70:fgf3*) embryos for 60 min at 18S/20S was sufficient to up-regulate *aldh1a3* expression exogenously in the dorsomedial part of the OV, indicating that FGF is upstream of *aldh1a3* expression, but that only part of the OV is competent to respond (Fig. 9M–P). The sufficiency of FGF for ectopic otic *aldh1a3* expression was even seen with heat shock at 20S, arguing strongly that the requirement for FGF for endogenous *aldh1a3* expression is independent of early FGF functions in otic induction or anteroposterior patterning. Although this result predicts an epistatic relationship, indicating that RA inhibition would not exacerbate the otic phenotype caused by loss of FGF function, we found that simultaneous treatment with both FGF and RA inhibitors (10 µM SU5402, 300 µM DEAB) resulted in whole embryo developmental arrest and death by 24 hpf. We were therefore unable to examine the effects of combined inhibition of FGF and RA at OV stages on otic patterning.

To test whether FGF expression is dependent on RA signalling, we manipulated RA signalling and analysed expression of *fgf3* and *fgf8a*, together with the FGF target gene *dusp6*, at 26 hpf. Exposure of embryos to 10 nM RA decreased otic *fgf3, fgf8a* and *dusp6* expression (Fig. 9R,U,X), whereas treatment with the RA antagonist DEAB resulted in a modest expansion of the otic *fgf3*, *fgf8a* and *dusp6* expression domains in the medial and posterior OV floor (Fig. 9S,V,Y). For both treatments, only a single stripe of *fgf3* expression in the pharyngeal pouch mesenchyme remained (Fig. 9S–T). Heat-shock of Tg(*hsp70*:*dnrar*) embryos at 18S resulted in the up-regulation of *fgf3* expression in a subset of otic cells, which we assume reflects the mosaic expression of the transgene (Fig. 9W–Y). Taken together, our results suggest that while FGF activity regulates RA production positively (via *aldh1a3* expression), RA itself acts to down-regulate FGF activity. This raises the interesting possibility that FGF and RA signalling form a feedback loop in the anterior OV that is required for the correct patterning of the ventral OV.

### RA signalling has FGF-dependent and independent functions during otic vesicle development

**Figure 10:**
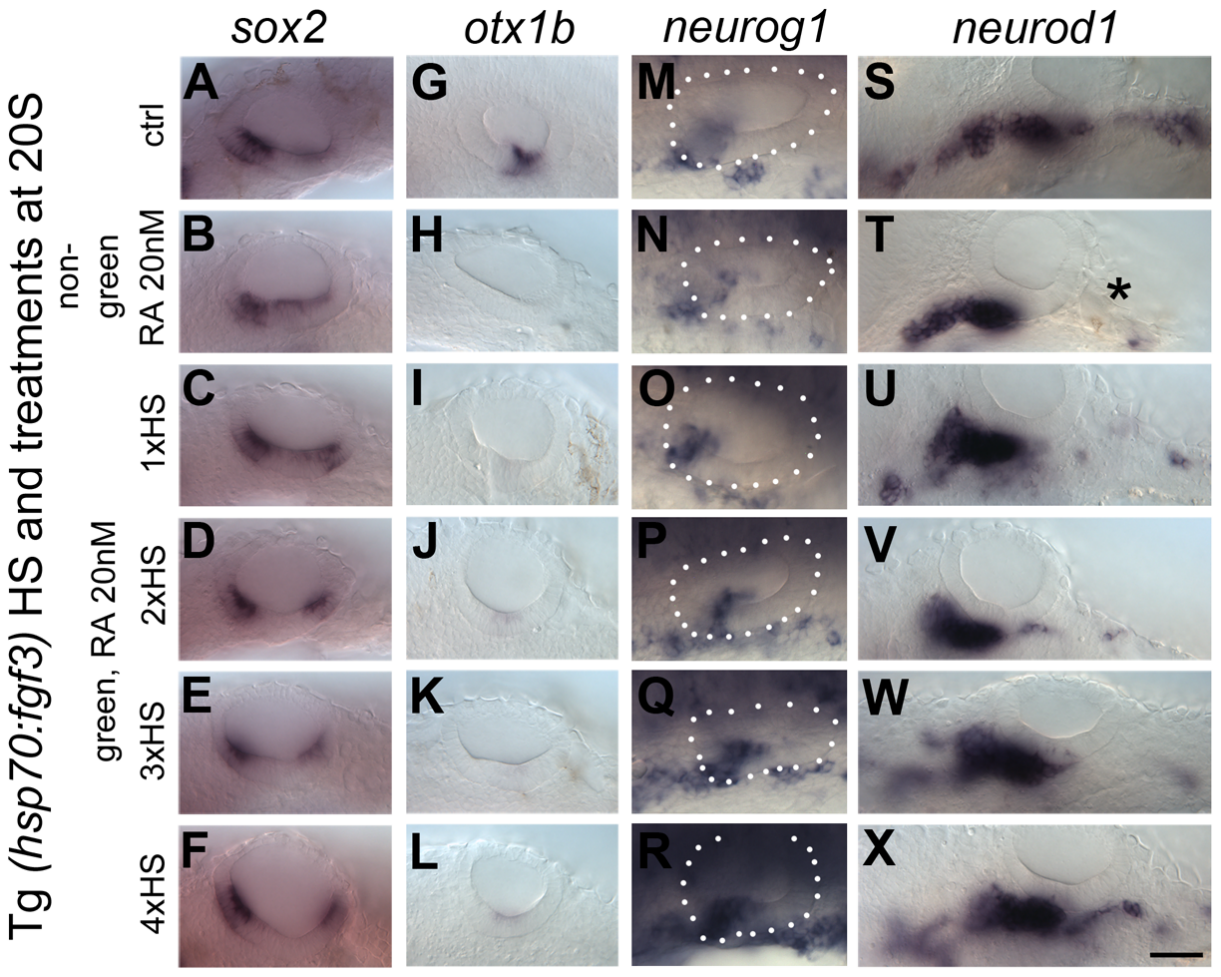
Elevated levels of FGF3 can counteract the effect of elevated RA signalling on sensory development. Tg(*hsp70:fgf3*) embryos were treated with DMSO (A,G,M,S) or 20 nM RA from 20S and the first heat shock applied at the same time. Embryos were heat shocked never (A,G,M,S), once (1×HS; C,I,O,U), twice (2×HS; D,J,P,V), three times (3×HS; E,K,Q,W) or four times (4×HS; F,L,R,X) and sorted as non-green non-transgenic siblings (B,H,N,T) or green transgenic embryos (C–X). Non-green non-transgenic siblings were pooled; different HS treatments did not affect the phenotype (B,H,N,T). (A,G,M,S) Tg(*hsp70:fgf3*) embryos treated with DMSO show normal expression of the sensory marker *sox2* (A) and the non-neurogenic marker *otx1b* (G) and the otic markers *neurog1* (M) and *neurod1* (S). (B) In non-green sibling embryos treated with 20 nM RA, the expression of *sox2* extends across the ventral OV floor. (C–F) In Tg(*hsp70:fgf3*) embryos treated with 20 nM RA and given 1×HS (C) or 2×HS (D), the expression of *sox2* is present in two extended domains at the anterior and posterior of the OV. (E) In Tg(*hsp70:fgf3*) embryos treated with 20 nM RA and 3×HS the expression of *sox2* is normal or slightly reduced. (F) In Tg(*hsp70:fgf3*) embryos treated with 20 nM RA and 4×HS the expression of *sox2* is slightly reduced or normal; the posterior domain of expression is shifted laterally. (H) In non-green sibling embryos treated with 20 nM RA, the expression of *otx1b* is reduced. (I–L) In Tg(*hsp70:fgf3*) embryos treated with 20 nM RA and given 1×HS (I), 2×HS (J), 3×HS (K) or 4×HS (L), the expression of *otx1b* is greatly reduced. (N) In non-green sibling embryos treated with 20 nM RA, the expression of *neurog1* is reduced in the OV. (O,P) In Tg(*hsp70:fgf3*) embryos treated with 20 nM RA and given 1×HS (O) or 2×HS (P), the expression of *neurog1* is reduced in the OV. (Q) In Tg(*hsp70:fgf3*) embryos treated with 20 nM RA and 3×HS the expression of *neurog1* is normal but slightly shifted towards posterior. (R) In Tg(*hsp70:fgf3*) embryos treated with 20 nM RA and 4×HS the expression of *neurog1* is increased but shifted to a more posterior position in the floor of the OV. (S,T) In non-green sibling embryos treated with 20 nM RA, the expression of otic *neurod1* is slightly increased, while expression is lost in prospective posterior lateral line and vagal ganglion cells posterior to the OV (asterisk). (U–W) In Tg(*hsp70:fgf3*) embryos treated with 20 nM RA and given 1×HS (U), 2×HS (V) or 3×HS (W) the expression of *neurod1* is increased in the OV. In embryos heatshocked three times neuroblasts emerge in a more posterior position in the OV floor (W). (X) In Tg(*hsp70:fgf3*) embryos treated with 20 nM RA and 4×HS the expression of *neurod1* is increased and otic neuroblasts emerge from a more posterior position in the floor of the OV. In addition, *neurod1*-positive cell populations re-emerge below the OV. All panels are lateral views with anterior to the left. For numbers see Table 1. Scale bar: 50 µm.

Since exogenous RA can inhibit expression of *fgf3* in the OV and influences sensory development in a manner similar to low level FGF inhibition with SU5402 or *lia* (*fgf3^−/−^*) mutant phenotypes, we wanted to test if the results obtained through RA treatment are caused simply by inhibiting *fgf3* expression and thus lowering FGF levels. If that were the case, raising levels of FGF3 in RA-treated embryos should rescue the observed phenotype. Tg(*hsp70:fgf3*) embryos were treated with 10 nM (Table 1) or 20 nM RA (Fig. 10; Table 1) from 18S and immediately heat-shocked once, twice, three or four times for 1 hour to generate embryos with different levels of FGF3 rescue, and stained for *sox2, otx1b*, *neurog1* and *neurod1*. While in 95–100% of RA-treated control (heat shocked, non-green) embryos, the anterior and posterior sensory patch marked by *sox2* was closer together or fused, fewer (1×HS, 2×HS) or no (3×HS, 4×HS) embryos displayed this phenotype in the transgenic heat-shocked siblings that over-express *fgf3* (Fig. 10A–L; Table 1). Instead, the two *sox2* patches were now more widely spaced, and the posterior patch was reduced in size. This resembles the phenotype generated by 1×HS of Tg(*hsp70:fgf3*) embryos without RA treatment, which we designated ‘Fgf3-like’ (Table 1; Fig. 4). These data suggest that RA acts on *sox2* expression through its ability to influence *fgf3* expression. By contrast, in RA-treated, heat-shocked green embryos, compared with RA-treated non-green embryos, elevated levels of FGF signalling were not sufficient to override the suppressive effect RA signalling has on *otx1b* expression (Fig. 10M–R; compare with Fig. 4). These results indicate that RA can down-regulate *otx1b* expression directly, independent of its inhibitory effect on FGF signalling.

**Table 1:** Distribution of *sox2* phenotypes after RA and FGF manipulation.

	Otic *sox2* expression pattern				
Treatment	wild-type	RA-like (closer, fused sensory patches)	FGF3-like (widely-spaced patches, reduced P patch)	abnormal embryo	*n*
Control (untreated, non-transgenic)	100%	-	-	-	22
10 µM RA, non green	-	95%	-	5%	19
10 µM RA, 1×HS	58%	28%	14%	-	30
10 µM RA, 2×HS	56%	23%	10%	11%	9
10 µM RA, 3×HS	30%	-	50%	20%	10
10 µM RA, 4×HS	33%	-	56%	11%	9
Control (untreated, non-transgenic)	100%	-	-	-	21
20 µM RA, non green	-	100%	-	-	21
20 µM RA, 1×HS	84%	4%	4%	8%	25
20 µM RA, 2×HS	59%	17%	24%	-	17
20 µM RA, 3×HS	53%	-	47%	-	17
20 µM RA, 4×HS	25%	-	75%	-	16

Abbreviations: HS, heat shock of Tg(*hsp70:fgf3*) embryos; *n*, number of embryos; RA-like, similar phenotype to RA treatment alone (closer or fused *sox2*-expressing sensory patches); FGF3-like, similar phenotype to FGF heat shock alone, without RA treatment (widely spaced *sox2*-expressing sensory patches, with a reduction in the posterior patch).

Both FGF and RA signalling have a positive effect on emerging *neurod1*-positive otic neuroblasts [12], [14] (Figs 4, 8, 10) but while exogenous RA has little effect on otic *neurog1* expression (Fig. 10M,N; S8 Figure), *neurog1* is up-regulated in embryos with elevated FGF3 levels (Fig. 4Y,Z). Expression of *neurog1* is up-regulated in embryos treated with 20 nM RA and higher levels of *fgf3* over-expression (3×HS, 4×HS) (Fig. 10Q,R). Expression of otic *neurod1* is increased in all combinations of FGF and RA compared with embryos treated with RA alone (Fig. 10S–X). Note that RA signalling in this context leads to a loss of *neurod1* expression in a domain posterior to the ear, likely to include posterior lateral line and vagal ganglia (Fig. 10), which is expanded in embryos with elevated *fgf3* levels alone (Fig. 4). The increase in *neurod1*-positive otic neuroblasts in embryos with both RA treatment and elevated *fgf3* expression probably reflects an enhancement of the *neurog1*-positive pool by FGF3 and a drive to neuroblast maturation (expression of *neurod1*) by RA signalling.

## Discussion

We have analysed signalling requirements in patterning the ventral floor of the zebrafish otic vesicle (OV), which gives rise to sensory, neuronal and non-neural fates. We provide evidence that FGF signalling is required for normal OV development at a time point after its function in otic placode induction and anterior patterning. In the OV, FGF3 is required to restrict sensory fates and is also both required and sufficient to promote expression of the non-neural marker *otx1b* in the ventral floor of the OV. RA signalling has the opposite role, promoting sensory fates and restricting *otx1b* expression and the development of non-neural territories. Both RA and FGF, however, are required for the development of the neurogenic domain and formation of the correct number of neuroblasts. We have identified separate roles for FGF3 and FGF8A in ventral OV patterning. Moreover, our results indicate that FGF and RA signalling form a feedback loop in the OV.

### Specification of the non-neural (*otx1b*-expressing) region

We have identified FGF signalling as a key regulator of *otx1b* expression and thus non-neural otic ventral floor fates, including the ventral pillar of the lateral semicircular canal duct. *otx1b* is regulated by Tbx1: expression of *otx1b* is lost in the *tbx1* (*vgo*) mutant [7] and both *tbx1* mutant (*vgo*) and *otx1b* mutant or morphant embryos display an expansion of the neurogenic domain and a loss of non-neural ventral otic floor identities [2], [6], [28] (and this work). Embryos treated with the FGF inhibitor SU5402 from 18/20S onwards until 26 hpf lose the expression of *tbx1* and *otx1b*, indicating the loss of non-neural ventral fates. In comparison, treatment with 10 µM SU5402 from earlier stages (10S for 3 h (to 16S) or 5 h (to 20S)) does not affect otic *otx1b* expression; likewise, the ventral pillar is still present under these conditions [12]. This indicates that FGF signalling after the 18S stage is required for correct development of the non-neural domain of the ventral OV floor.

During otic placodal stages (10.5 hpf in the zebrafish, 9 hours before the treatments in our study at 20S) mesodermal RA emanating from *aldh1a2/Raldh2*-expressing cells is required to specify *tbx1/Tbx1*-positive cells in zebrafish, chick and mice [2], [29], promoting posteroventral otic identities. Since protein synthesis is not required for this process in chick, this seems to be a direct regulatory effect exerted by RA on *Tbx1* [30]. Interestingly, an expansion of *Tbx1* expression in chick otocysts was only observed when a RA bead was implanted at E1.5 (∼HH10, 10S) but not at E2 (HH13, 19S) [29], suggesting a narrow temporal window in which RA can influence *Tbx1* expression directly. This change in RA-responsiveness precedes the onset of *Raldh3* expression in the chick otocyst itself at HH18 by 24 h [31], suggesting later non-described functions of RA signalling during inner ear development.

Our results indicate that RA signalling emanating from the *aldh1a3* expression domain in the anterior OV acts at later stages of inner ear development in the zebrafish. Exogenous RA application at these later stages inhibits the expression of *otx1b*, but not *tbx1*, suggesting a Tbx1-independent restriction of *otx1b* by RA. Moreover, abrogation of RA signalling at these later stages (by chemical inhibition, over-expression of dnRAR or morpholino-mediated *aldh1a3* knockdown) only slightly alters *tbx1* expression in the zebrafish ear, while *otx1b* expression is expanded anteriorly. *aldh1a3* expression in the OV starts at 22S, slightly after the onset of *otx1b* expression; both *aldh1a3* and *otx1b* depend on FGF signalling for their expression. This suggests that RA signalling through *aldh1a3* at the OV stage is required in the anterior OV to restrict the anterior expansion of *otx1b* expression induced by FGF, thus regulating the position of the neural/non-neural boundary in the ventral OV floor. In medaka, however, *aldh1a3* has been lost from the genome, and *aldh1a2* takes over the ventral expression and subfunction of *aldh1a3* in the developing eye [32]. It will be interesting to see whether there is a similar replacement by *aldh1a2* in medaka of the specific role for *aldh1a3* in ear patterning that we have uncovered here for the zebrafish.

### Specification and spacing of the sensory maculae

An early sign of sensory differentiation in the zebrafish inner ear is the expression of *atoh1b* from otic placodal stages, marking the differentiation of pairs of tether cells at the anterior and posterior poles of the OV [26]. Perturbing FGF or RA signalling at the OV stages results in abnormal sensory patch development and spacing, suggesting that even though differentiation has been initiated, not all cells in and outside the sensory region are fully committed. If FGF signalling is abolished completely, only a few hair cells develop and sensory markers are down-regulated or lost, indicating that FGF signalling is required for sensory development, in line with results from the Riley group [26].

Interestingly, in our study, treatment with low and intermediate levels of SU5402 resulted in the up-regulation of *sox2* expression and the development of supernumerary hair cells at later stages after relief from FGF inhibition, mimicking the result observed when embryos are treated with RA. Thus transient low level FGF inhibition or exogenous activation of the RA signalling pathway both lead to an expansion of the sensory domain. In the zebrafish, expansion of the sensory region occurs in a spatially restricted manner, along the ventromedial OV. This is likely to correspond to the region of sensory competence thought to be marked by *dlx3b/4b* expression in the placodal ectoderm [33], which could explain the spatial preference in sensory expansion that we observe with either partial FGF inhibition or increased RA signalling.

Analysis of sensory markers in embryos mutant for either *fgf8a (ace)* or *fgf3 (lia)* reveals a differential requirement for these FGFs in zebrafish OV development. While sensory development is reduced in *fgf8a (ace)* mutant embryos, the two sensory patches appear fused in *fgf3 (lia)* mutants and the posterior patch is enlarged, suggesting a re-specification of the inter-sensory patch epithelium. In embryos with elevated levels of FGF3 from the 18/20S stage, the anterior part of the posterior patch is missing, strengthening the notion that FGF3 is sufficient to suppress sensory fate specification in ventromedial otic regions. FGF3 and FGF8A belong to two different FGF subfamilies and preferentially signal through specific FGFR isoforms [34]. In zebrafish, our data indicate that *fgfr4* is mainly expressed in the sensory region of the OV at 26 hpf; FGF8A but not FGF3 signals preferentially through FGFR4 [23], further supporting the non-redundant functions of different FGF ligands in OV development. FGF3 signalling has been implicated in non-sensory development in mouse. In mice mutant for the FGF3- and FGF10- specific FGFR2(IIIb) isoform [23], which is expressed in the non-sensory epithelia of the otocyst, inner ear development initially occurs normally, but morphogenesis is disturbed [35].

In mice, several studies have implicated FGF signalling in cell fate decisions in the cochlea [36], [37], [38], [39], [40], [41]. In the auditory sensory epithelium, FGF8 is expressed in the earliest differentiating cells, the inner hair cells, where it controls differentiation of the adjacent pillar cells [37], [38], [39]. In zebrafish, a model has been proposed in which FGF8A can confer sensory competence to cells in the OV when over-expressed ectopically together with *atoh1a* [27]. Our results suggest that the role FGF signalling plays in the context of otic sensory development is more diverse. High levels of FGF signalling suppressed the acquisition of sensory fates, while lowering endogenous levels of FGF signalling from 18/20S onwards was sufficient to induce sensory fates, without additional over-expression of *atoh1a*.

RA signalling can inhibit otic *fgf3* and *fgf8a* expression, with application of exogenous RA resulting in a similar expansion of sensory fates in the zebrafish ear as that seen with lowered FGF signalling. Our results show that elevated levels of FGF can rescue the sensory expansion induced by RA, indicating that RA acts through lowering FGF levels in this case. Nevertheless, inhibition of RA signalling reveals that RA is required for sensory patch development and hair cell differentiation. The posterior patch responds more strongly to lowered levels of endogenous RA, and we speculate that this might be because endogenous RA levels at around 1 dpf are lower at the posterior pole of the OV, and thus inhibition here might be more complete. Again, loss of RA signalling shows similarities to a gain in FGF signalling. We have shown that down-regulation of RA signalling can lead to an expansion of *fgf3* and *fgf8a* expression in the OV. This suggests that RA might be either directly required in the sensory lineage or might act indirectly through its FGF regulatory function. Taken together, our data suggest that endogenous levels of RA and FGF signalling have to be tightly regulated for correct sensory development to occur, and only slight perturbations can have significant effects for overall ear morphology and function.

### Specification and restriction of the neurogenic region

Inhibition of RA signalling at OV stages decreases the otic *neurog1*- and *neurod1*-positive domain dramatically. The decrease is even more pronounced with RA signalling inhibition compared with FGF signalling inhibition in our hands. The role of FGF in otic neurogenesis is well established [8], [13], [14], but our results indicate that RA signalling is also required in the otic neurogenic lineage at later stages of OV development. Because *otx1b* expression is expanded in embryos in which RA signalling is inhibited, the action of RA on neurogenesis might be direct or indirect. Embryos mutant for *otx1b* have an expanded *neurod1*-positive domain, suggesting that Otx1b has a role in limiting neurogenesis. Support for a direct role of RA signalling in otic neuroblast development comes from our analysis of *neurod1* expression in *tbx1^−/−^ (vgo)* mutant embryos. Here, otic *otx1b* expression is absent, but RA inhibition still leads to a decrease in *neurod1* expression, suggesting that RA might act directly in the neurogenic domain. Embryos with elevated levels of RA signalling have a slightly decreased *neurog1*-positive domain in the OV, while both the *neurod1*- and the *isl1*-positive domains are increased. This suggests that RA acts in the neurogenic lineage by driving *neurod1* expression prematurely, depleting the *neurog1*-positive pool of specified progenitor cells. In all our treatments, we cannot distinguish unequivocally between an effect on size of the neurogenic domain within the epithelium, and an effect on neuroblast proliferation after cells have left the otic epithelium. Impaired neuronal development has also been reported in mice embryos double mutant for RARα/RARγ, in which the VIIIth ganglion is hypoplastic [42].

### Feedback and integration of the FGF and RA signalling pathways in the zebrafish ear

Both FGF and RA signalling are implicated in the earliest phases of otic development, including otic induction, which in zebrafish occurs at around 11–14 hpf. We have identified a time window for their requirement beginning five hours later, at the 20S stage, coinciding approximately with the onset of *otx1b* expression in the non-neural regions of the ventral OV. Soon afterwards, at around 22S, *aldh1a3* starts to be expressed in the anterior ventral OV. Both *otx1b* and *aldh1a3* depend on ongoing high levels of FGF signalling, but RA signalling is sufficient to restrict both FGF signalling from reaching too far posteriorly and *otx1b* expression from spreading too far anteriorly, providing a beneficial signalling niche for the emerging otic neuroblasts.

**Figure 11:**
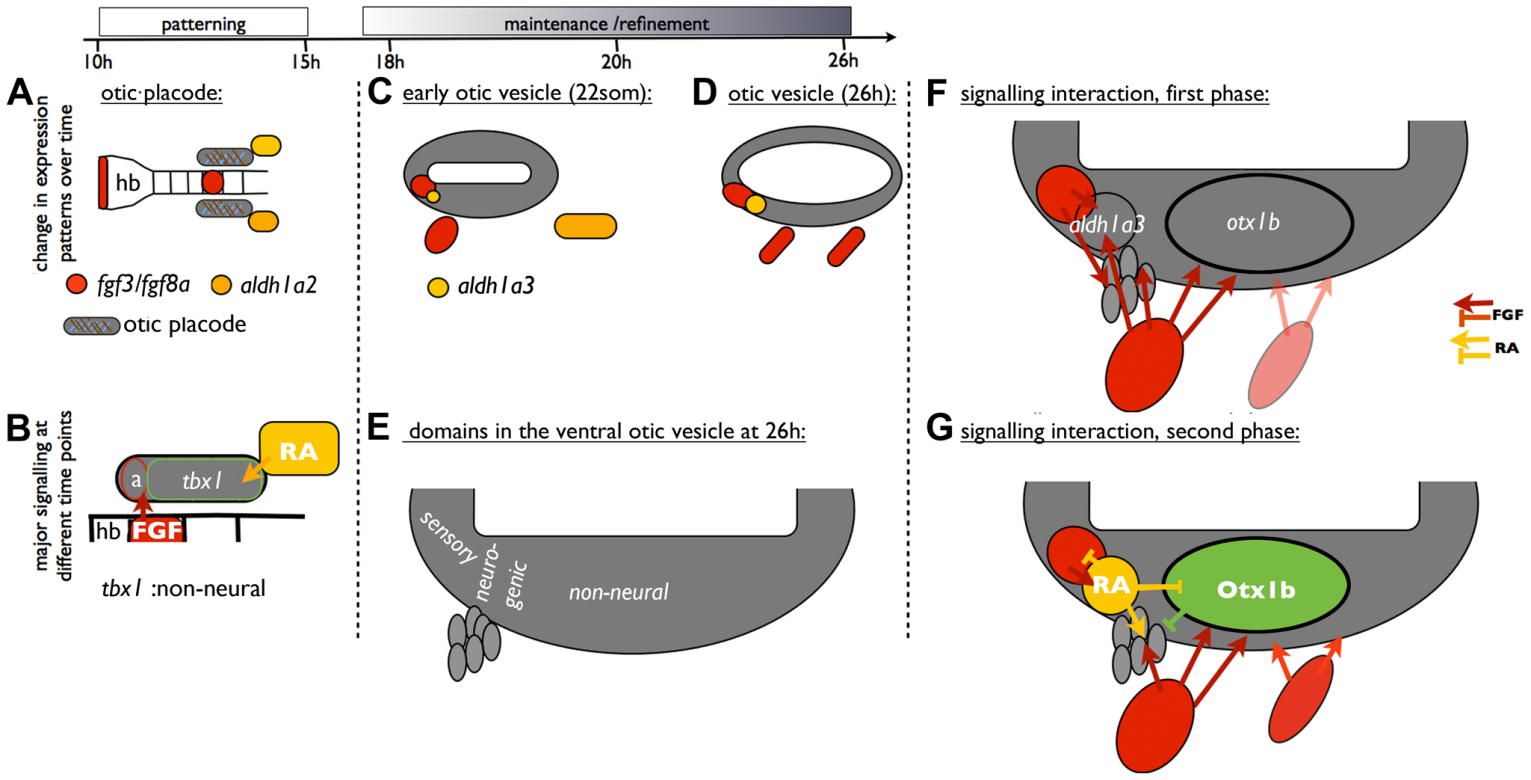
A model of ventral otic patterning in the zebrafish. (A,B) At otic placode stages, FGF3 from the hindbrain patterns anterior otic regions, while RA signalling is required for the expression of *tbx1* and subsequent non-neural development (based on [2], [12]. (C–E) At OV stages, FGF and RA signalling pathway members contribute to maintenance of otic patterning. It is likely that both otic and pharyngeal *fgf3* expression sources influence otic patterning, although our experiments have not distinguished these. In addition, expression of *otx1b* in the OV starts from around 18S. (F) FGF signalling is required for the expression of *aldh1a3* and *otx1b* in the OV. (G) Cells expressing *aldh1a3* in the OV itself provide a local source of RA. RA signalling acts in a negative feedback loop to restrict *fgf*-expressing cells anteriorly. In addition, RA signalling prevents *otx1b* expression from spreading into anteroventral neurogenic regions of the OV and promotes the maturation of otic neuroblasts. Abbreviations: a, anterior otic placode; hb, hindbrain.

Taken together, our results support the following model of OV patterning and refinement (Fig. 11): *fgf3* expression is required for the normal otic expression of *otx1b* from around 20S. High FGF signalling induces *aldh1a3* expression in an anterior, ventromedial position in the OV from around 22S. Elevated levels of FGF3 are sufficient to up-regulate *aldh1a3* expression in the dorsal and intermediate OV, suggesting that these regions are competent to respond to FGF. As development proceeds, the cells expressing *aldh1a3* in the OV itself provide a local source of RA. RA signalling could then act in a negative feedback loop to restrict *fgf3-* and *fgf8a-*expressing cells anteriorly. In mice, the negative regulatory effect exogenous RA exerts on *Fgf3* is well established [9], [43], [44], and in mouse embryos mutant for the RA-degrading enzyme CYP26, elevated RA levels repress *Fgf8* expression [45]. In addition, RA emanating from the somites has been shown to inhibit FGF signalling in emerging spinal cord [46] and forebrain [47]. While FGF8 emerging from the pre-neural tube inhibits *Raldh2* expression in the adjacent presomitic mesoderm, RA in turn down-regulates *Fgf8* expression [46].

Both FGF and RA signalling pathways play a positive role in neuroblast formation in the OV. Inhibition of either FGF or RA signalling decreases *neurod1* expression. One idea is that, independent from its function to repress the anterior spread of *otx1b* expression, RA is also required for neuroblast maturation in the statoacoustic ganglion. It has been suggested that opposing actions of FGF and RA signalling control the timing of the epithelial-mesenchymal transition in trunk neural crest cells and consequently emigration of NCC from the neural tube [48]. It will be interesting to see whether FGF and RA signalling have a similar function during neuroblast emigration from the OV.

## Materials and Methods

### Ethics statement

All animal experiments were performed under licence from the UK Home Office and passed University of Sheffield local ethical review.

### Animals

Zebrafish lines used were AB, London wild-type (LWT), *ace^ti282a^* (*fgf8a^−/−^*) [24], [49], *lia^t21142^* (*fgf3^−/−^*) [25], *nac^w2/w2^* (*mitfa^−/−^*) [50], *nls* (*aldh1a2^−/−^*) [18], *otx1b^sa96−/−^* (Sanger Institute Zebrafish Mutation Resource), *vgo^tm208^* (*tbx1^−/−^*) [7], [51], Tg(*hsp70l:dnraraa-EGFP*) (*pd18Tg*), hereafter referred to as Tg(*hsp70:dnRAR*) [52], Tg(*hsp70:fgf3*) [53] and Tg(*pou4f3:gfp*) [54].

### Chemical treatment, heat shock and embryo culture conditions

Embryos were raised in E3 medium (5 mM NaCl, 0.17 mM KCl, 0.33 mM CaCl_2_, 0.33 mM MgSO_4_, 0.0001% methylene blue). Embryonic stages are given as hours post fertilisation (hpf) at 28.5°C or as somite stages (S) [55], [56]. Embryos were dechorionated prior to chemical treatment. Embryos were treated with 5, 10,15 or 20 µM SU5402 (Calbiochem), 5, 10 or 20 nM retinoic acid (RA, Sigma) or DEAB (Alfa Aesar, 2 year old batch) in DMSO or with DMSO alone. To titrate our batch of DEAB we repeated a published experiment with 20 µM, 50 µM, 100 µM, 200 µM or 400 µM DEAB: we found 400 µM to block RA signalling efficiently and used this concentration (S6 Figure). The age of our DEAB batch may explain why our treatments required higher concentrations than those previously published. DMSO volume was always matched to that used for the highest experimental treatment.

Tg(*hsp70:dnRAR*) transgenic and sibling embryos from a Tg(*hsp70:dnRAR/+*)×Tg(*hsp70:dnRAR/+*) or a Tg(*hsp70:dnRAR/+*)×*nac/nac* cross were incubated at 39°C for 45 min. Transgenic and sibling embryos were distinguished by the expression of GFP. Because the Tg(*hsp70:dnRAR*) line drives transgenic expression in a mosaic manner, transgenic embryos were sorted as ‘weak green’, ‘green’ and ‘very green’ depending on GFP expression. Only ‘green’ and ‘very green’ embryos were used for further analysis (see S7 Figure). Tg(*hsp70:fgf3*) transgenic and sibling embryos from a Tg(*hsp70:fgf3/+*)×Tg(*hsp70:fgf3/+*) cross were incubated at 38°C for 1 hour (1×HS). To increase the strength of the heat shock, embryos were allowed to cool down for 30 min before repeating the procedure two, three or four times (2×HS, 3×HS and 4×HS). Transgenic Tg(*hsp70:fgf3*) embryos were identified by their green hearts at 24 hpf.

### Morpholino injection

To knock down *aldh1a3*, we used a previously published translation-blocking morpholino: 5′-TATAGTCCCGTTCTGTGCCATAGCA-3′ [57]. The morpholino was dissolved in water (with fluorescent dextran added for visibility) and injected at 1 mM into one- or two-cell stage embryos (1–1.5 nl per injection). Controls were uninjected stage-matched sibling embryos and sibling embryos injected with a matched volume of water and fluorescent dextran alone (‘mock-injected’) to exclude injection artifacts.

### *In situ* hybridisation

Single or double whole-mount *in situ* hybridisation was carried out as described [58], [59]. Probes used were *aldh1a2* [18], *aldh1a3* [19], *atoh1a* [60], *cyp26c1* [20], *dusp6* [61], *etv4* [62], *fgf3* [63], *fgf8a* [24], *fgfr1a, fgfr3, fgfr4* [64], *fgfr2* [65], *isl1* [66], *neurod1* [67], *neurog1* [68], *otx1b* [3], *raraa, rarab, rarga, rargb* [69], *sox2* [70], *tbx1* [51], and *tecta* (G. Stooke-Vaughan and TTW, unpublished data).

### FITC-Phalloidin staining

Staining was carried out as described [71].

### Live imaging and cell counting

Embryos were anaesthetised in 0.5 mM MS222 (3-aminobenzoic acid ethyl ester) and mounted in 3% methylcellulose. Tg(*pou4f3:gfp*) embryos or FITC-Phalloidin stained embryos were imaged with a 20× objective on an Olympus FV-1000 confocal microscope. Fiji was used to adjust brightness and contrast and to count cells. Cell count data were analysed using GraphPad Prism.

### *n* numbers

For chemical treatments and morpholino experiments, we have indicated the number of embryos treated or injected (e.g. *n* = 10). If the phenotype was not consistent, the number of embryos showing the phenotype out of the total is given (e.g. *n* = 9/10). For analysis of batches of embryos from a cross between heterozygous carriers of a mutation, we have indicated the numbers of siblings (wild-type or heterozygous, e.g. *n* = 9/12) and the presumed homozygous mutants (e.g. *n* = 3/12) that show an expression pattern or phenotype. If all embryos showed the same phenotype, the number indicates the number of embryos used (e.g. *n* = 12).

## Supporting Information

S1 Figure Expression of Fgf and RA receptor genes in the zebrafish otic vesicle. (A–L) Expression of FGF receptor genes in the zebrafish OV at 22S and 26 hpf. (A–D) *fgfr1a* is expressed fairly ubiquitously, including in the OV. (E–H) *fgfr2* is expressed in the posterior OV. (I–K) *fgfr4* is expressed in the medial OV at 22S. By 26 hpf expression becomes more restricted towards the poles and no expression can be detected in the ventral OV floor. (L) *fgfr3* is not expressed in the OV at 26 hpf. Vertical bars in B and F indicate position of section in D and H, respectively. (A,B,E,F,I,J) are dorsal views with anterior to the left. (C,G,K,L) are lateral views with anterior to the left; (D,H) are sections through the ear looking anteriorly. (M–P) Expression of RA receptor genes in the zebrafish OV at 26 hpf. (M) *raraa* is expressed very weakly in the anteroventral OV. (N) *rarab* is expressed in two patches in the OV. (O) *rarga* is expressed in most of the OV, but excluded from the anteroventral region, in a pattern very similar to that of *tbx1*. (P) *rargb* is expressed in two patches in the OV. (M,O,P) Lateral views; (N) dorsal view, anterior to the left. Scale bar: 50 µm.
(TIF)

S2 Figure Patterning of the otic vesicle in the *aldh1a2^−/−^ (neckless/nls)* mutant at 26 hpf. (A–E) Expression of non-neural markers *tbx1* (*n* = 14/19 from a heterozygous cross) and *otx1* (*n* = 39/52), the neuronal marker *neurod1* (*n* = 44/57), and the sensory markers *sox2* (*n* = 45/59) and *atoh1a* (*n* = 9/13) are normal in the OV of sibling embryos. (F–J) In *aldh1a2^−/−^ (nls)* mutants, patterning is relatively normal for all markers tested. Expression of *tbx1* (*n* = 5/19) and *otx1* (*n* = 13/52) is shifted slightly posteriorly, *neurod1* (*n* = 13/57) expression is slightly increased and shifted more posteriorly and expression of *sox2* (*n* = 14/59) and *atoh1a* (*n* = 4/13) is normal. All panels are lateral views with anterior to the left. Scale bar: 50 µm.
(TIF)

S3 Figure SU5402 efficiently down-regulates FGF target genes *etv4* and *dusp6*. Expression of the FGF-responsive genes *etv4* (A, *n* = 12) and *dusp6* (E, *n* = 6) is normal in embryos treated with DMSO from 18S to 26 hpf, but lost in embryos treated with 5 µM (B, *n* = 10; F, *n* = 8), 10 µM (C, *n* = 12; G, *n* = 10) and 15 µM (D, *n* = 9; H, *n* = 12) from 18S to 26 hpf. Inserts show the otic vesicle at higher magnification. All panels are lateral views with anterior to the left. Scale bars: 50 µm.
(TIF)

S4 Figure Wild-type expression pattern of sox2, atoh1a, tecta, neurod1, tbx1, otx1b, fgf3 and aldh1a3 in the zebrafish otic vesicle at 26 hpf. (A,B,G,H) *sox2* (purple) and *tecta* (red) are co-expressed in the presumptive anterior and posterior maculae. Expression of *sox2* (purple) is broader compared with expression of *tecta*. (C,D,I,J) *atoh1a* (weak purple) and *tecta* (red) are co-expressed in the presumptive anterior and posterior maculae. Expression of *atoh1a* (purple) is more restricted compared with expression of *tecta*. (E,F,K,L) The neurogenic marker *neurod1* (purple) is mainly expressed in neuroblasts of the statoacoustic ganglion beneath the OV; expression of *tecta* (red) marks the developing sensory maculae in the otic epithelium. (M,N,S,T) The non-neural marker *otx1b* (purple) and *tecta* (red) are expressed in distinct domains in the OV. Expression of *otx1b* can be detected in a ventrolateral domain. (O,P,U,V) *fgf3* (purple) and *tecta* (red) are co-expressed in the anterior OV. (Q,R,W,X) *aldh1a3* (purple) is expressed in the anterior OV, partially overlapping with the expression domain of *tecta* (red) but extending more ventromedially. (Y,B′) *aldh1a3* (purple) is expressed in the anterior OV in a position next to the expression domain of *neurod1* (red). (Z,A′,C′,D′) *tbx1* (purple) is expressed in the ventrolateral OV, posterior to the expression domain of *neurod1* (red). (E′) Schematic representation of the expression domains in relation to each other. a: anterior, p: posterior, d: dorsal, v: ventral, l: lateral, m: medial. Scale bar: 50 µm.
(TIF)

S5 Figure Expression of *otx1b* and *tbx1* in *lia (fgf3^−/−^)* and *ace (fgf8^−/−^)* mutant embryos. (A, D) Expression of *otx1b* is normal in *lia* sibling (*n* = 65/85 embryos from a heterozygous cross) and *ace* sibling (*n* = 64/91) embryos. (B) The expression domain and levels of *otx1b* are reduced in the *lia* mutant otic vesicle (*n* = 20/85). (C) The expression domain of *otxb1* is reduced in the much smaller ear of *ace* mutant (*n* = 27/91) embryos, but the overall pattern and level are normal. (E, H) Expression of *tbx1* is normal in *lia* sibling (*n* = 64/87) and *ace* sibling (*n* = 59/82) embryos. (F) Expression of *tbx1* is expanded anteroventrally in the OV of *lia* mutant (*n* = 23/87) embryos, filling in the anteroventral zone that is normally free of *tbx1* expression. (G) The pattern of *tbx1* expression is almost normal in *ace* mutant (*n* = 23/82) embryos, despite the smaller size of the OV. All panels are lateral views with anterior to the left. Scale bar: 50 µm.
(TIF)

S6 Figure Titration of the RA inhibitor DEAB. Note: this experiment repeats published data [2] where DEAB treatment from 10 hpf to 24 hpf resulted in a complete loss of otic *tbx1* expression. (A–F) Embryos were treated from 10 hpf to 24 hpf with DMSO (A) or varying concentrations of DEAB (B–F) and stained for *tbx1* at 24 hpf. (A) DMSO-treated embryos display a normal pattern of *tbx1* expression (*n* = 10). (B–D) No change in *tbx1* expression is detected in embryos treated with 20 µM (B, *n* = 62), 50 µM (C, *n* = 35) or 100 µM (D, *n* = 11) DEAB. (E) Expression of *tbx1* is down-regulated but not completely blocked in embryos treated with 200 µM DEAB (*n* = 13). (F) *tbx1* expression is blocked altogether in embryos treated with 400 µM DEAB (*n* = 14). (G–K) WT embryos treated with DMSO (G) or DEAB (H–K) at 18/20S, washed at 26 hpf and grown on to 55 hpf. (G) WT embryos treated with DMSO develop normally. (H,I) In WT embryos treated with 200 µM DEAB, ear development is fairly normal (H) or slightly perturbed (I). (J,K) In WT embryos treated with 400 µM DEAB, ear development is severely perturbed in most cases (90%; *n* = 9/10). The head is also reduced in size and embryos display pericardial oedema. All panels are lateral views with anterior to the left. Scale bar: 50 µm.
(TIF)

S7 Figure RA-inhibition: mosaic expression of GFP in Tg(hsp70:dnRAR) embryos and phenotype of DEAB-treated embryos at 55 hpf. (A–I) Embryos were heat shocked from 18S and pictures were taken at 26 hpf of embryos from one clutch of Tg(*hsp70:dnRAR*)♂ × *nacre* ♀. In all embryos, expression was mosaic in the otic region; representative embryos classified as ‘very green’, ‘green’ and ‘weak green’ are shown. All panels are lateral views with anterior to the left. Scale bar: 250 µm.
(TIF)

S8 Figure A role for RA in regulating zebrafish otic neurogenesis. Embryos were treated with DMSO, DEAB or RA from 18/20S to 26 hpf. (A–E) The dotted line demarcates the OV. Wild-type embryos treated with DMSO show normal expression of *neurog1* (A), while expression is decreased in the OV of wild-type embryos treated with DEAB (B). Otic expression of *neurog1* is relatively normal, or slightly reduced, in embryos treated with 5 nM (C), 10 nM RA (D) and 15 nM (E) RA. (F–J) Embryos treated with DMSO show normal expression of *neurod1* (F), while expression is decreased in the OV of embryos treated with DEAB (G), and increased in embryos treated with 5 nM (H), 10 nM (I) and 15 nM (J) RA. (K–O) Embryos treated with DMSO show normal otic expression of *isl1* (K), while expression is decreased in embryos treated with DEAB (L), and increased in embryos treated with 5 nM (M), 10 nM (N) and 15 nM (O) RA. All panels are lateral views with anterior to the left. Scale bar: 50 µm.
(TIF)
