# Supplementary figures and images for: RA and FGF Signalling Are Required in the Zebrafish Otic Vesicle to Pattern and Maintain Ventral Otic Identities
Source: PLoS Genet. 2014 Dec 4;10(12):e1004858. doi: 10.1371/journal.pgen.1004858 (PMC4256275; doi:10.1371/journal.pgen.1004858)

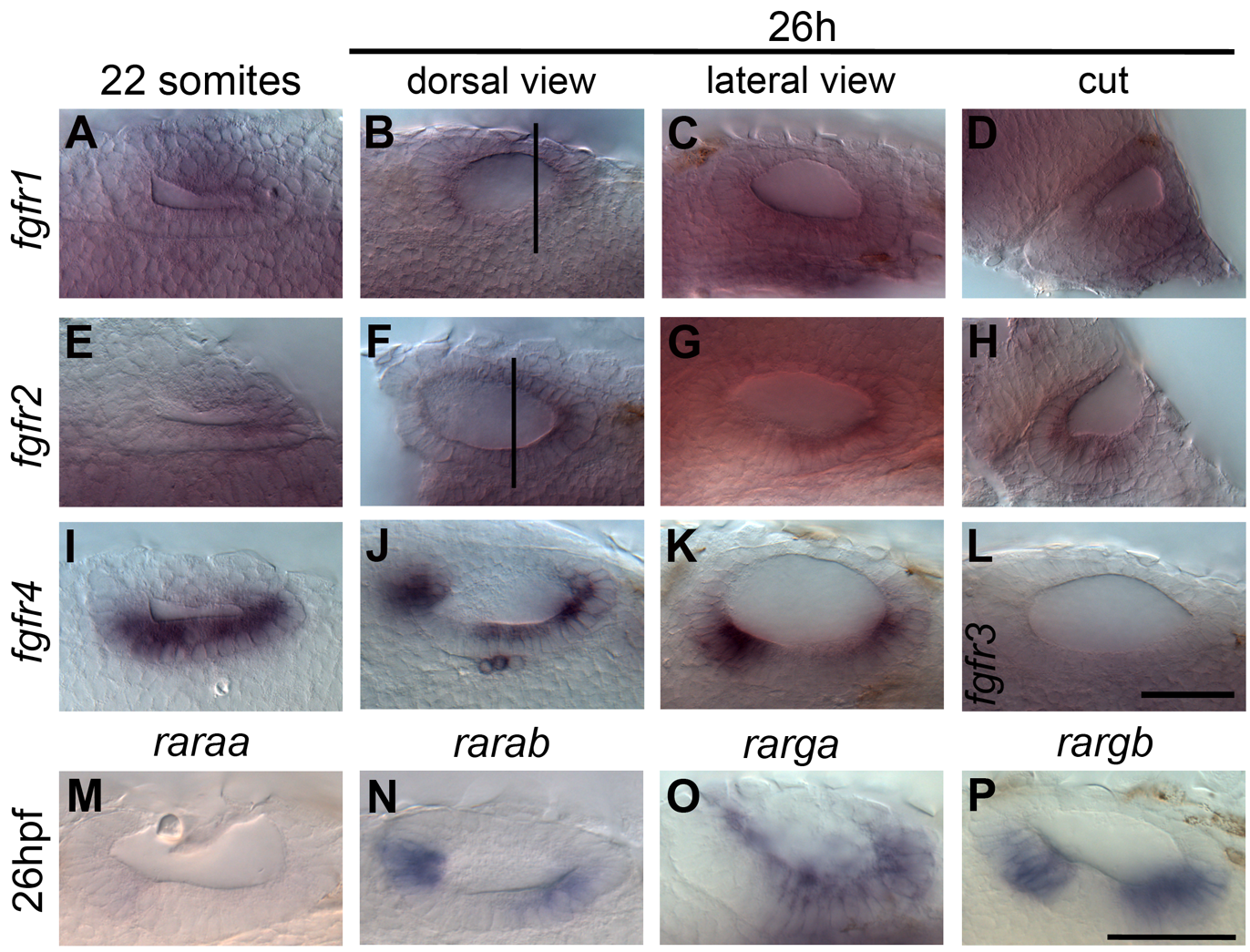

Supplement: S1 Figure — Expression of Fgf and RA receptor genes in the zebrafish otic vesicle. (A–L) Expression of FGF receptor genes in the zebrafish OV at 22S and 26 hpf. (A–D) fgfr1a is expressed fairly ubiquitously, including in the OV. (E–H) fgfr2 is expressed in the posterior OV. (I–K) fgfr4 is expressed in the medial OV at 22S. By 26 hpf expression becomes more restricted towards the poles and no expression can be detected in the ventral OV floor. (L) fgfr3 is not expressed in the OV at 26 hpf. Vertical bars in B and F indicate position of section in D and H, respectively. (A,B,E,F,I,J) are dorsal views with anterior to the left. (C,G,K,L) are lateral views with anterior to the left; (D,H) are sections through the ear looking anteriorly. (M–P) Expression of RA receptor genes in the zebrafish OV at 26 hpf. (M) raraa is expressed very weakly in the anteroventral OV. (N) rarab is expressed in two patches in the OV. (O) rarga is expressed in most of the OV, but excluded from the anteroventral region, in a pattern very similar to that of tbx1. (P) rargb is expressed in two patches in the OV. (M,O,P) Lateral views; (N) dorsal view, anterior to the left. Scale bar: 50 µm. (TIF) [file pgen.1004858.s001.tif]

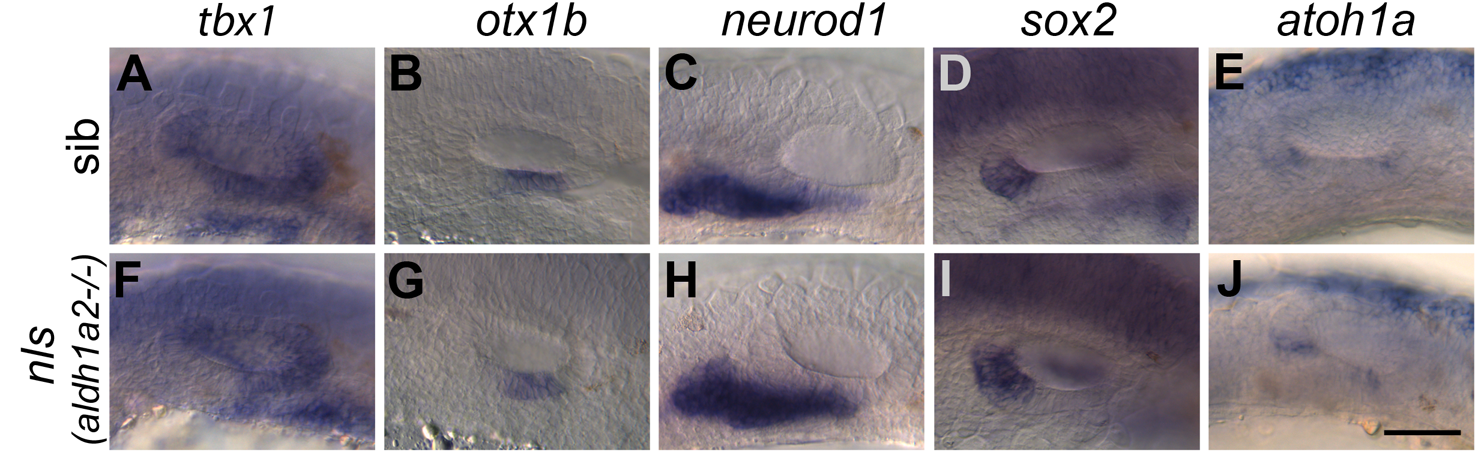

Supplement: S2 Figure — Patterning of the otic vesicle in the aldh1a2−/− (neckless/nls) mutant at 26 hpf. (A–E) Expression of non-neural markers tbx1 (n = 14/19 from a heterozygous cross) and otx1 (n = 39/52), the neuronal marker neurod1 (n = 44/57), and the sensory markers sox2 (n = 45/59) and atoh1a (n = 9/13) are normal in the OV of sibling embryos. (F–J) In aldh1a2−/− (nls) mutants, patterning is relatively normal for all markers tested. Expression of tbx1 (n = 5/19) and otx1 (n = 13/52) is shifted slightly posteriorly, neurod1 (n = 13/57) expression is slightly increased and shifted more posteriorly and expression of sox2 (n = 14/59) and atoh1a (n = 4/13) is normal. All panels are lateral views with anterior to the left. Scale bar: 50 µm. (TIF) [file pgen.1004858.s002.tif]

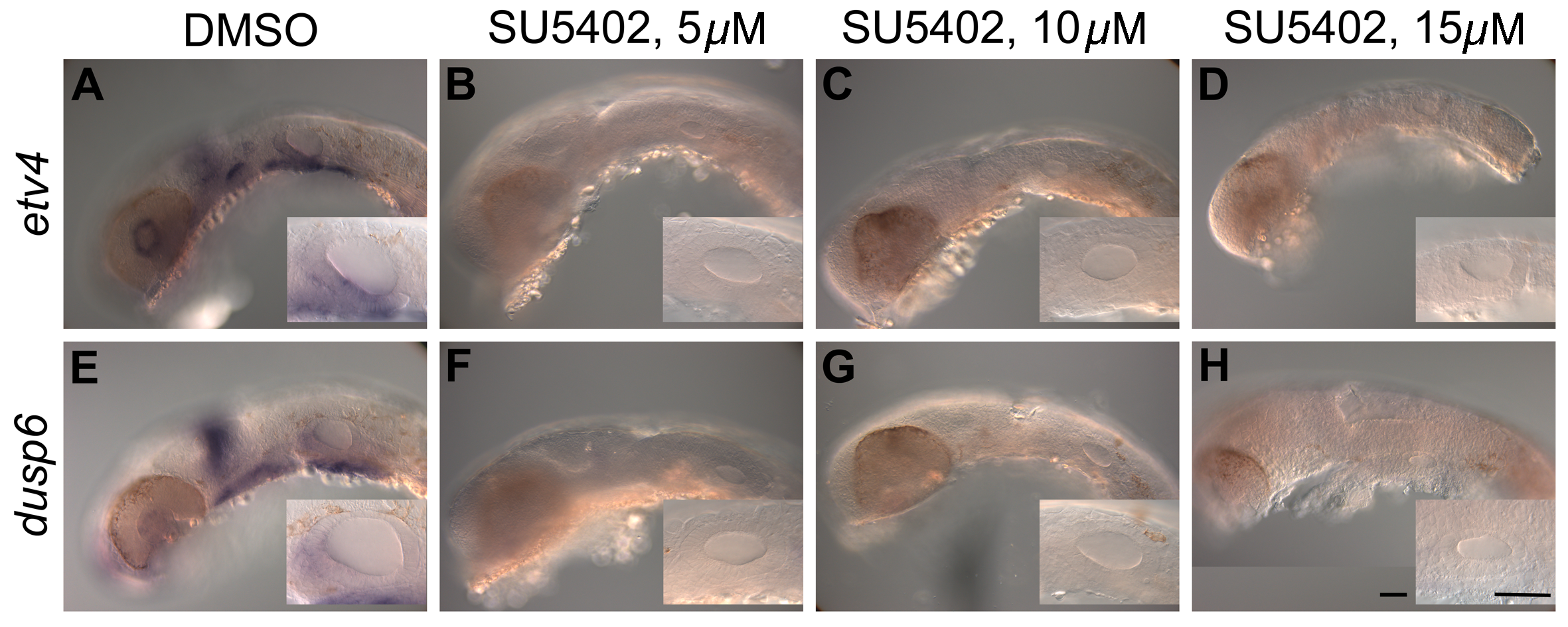

Supplement: S3 Figure — SU5402 efficiently down-regulates FGF target genes etv4 and dusp6. Expression of the FGF-responsive genes etv4 (A, n = 12) and dusp6 (E, n = 6) is normal in embryos treated with DMSO from 18S to 26 hpf, but lost in embryos treated with 5 µM (B, n = 10; F, n = 8), 10 µM (C, n = 12; G, n = 10) and 15 µM (D, n = 9; H, n = 12) from 18S to 26 hpf. Inserts show the otic vesicle at higher magnification. All panels are lateral views with anterior to the left. Scale bars: 50 µm. (TIF) [file pgen.1004858.s003.tif]

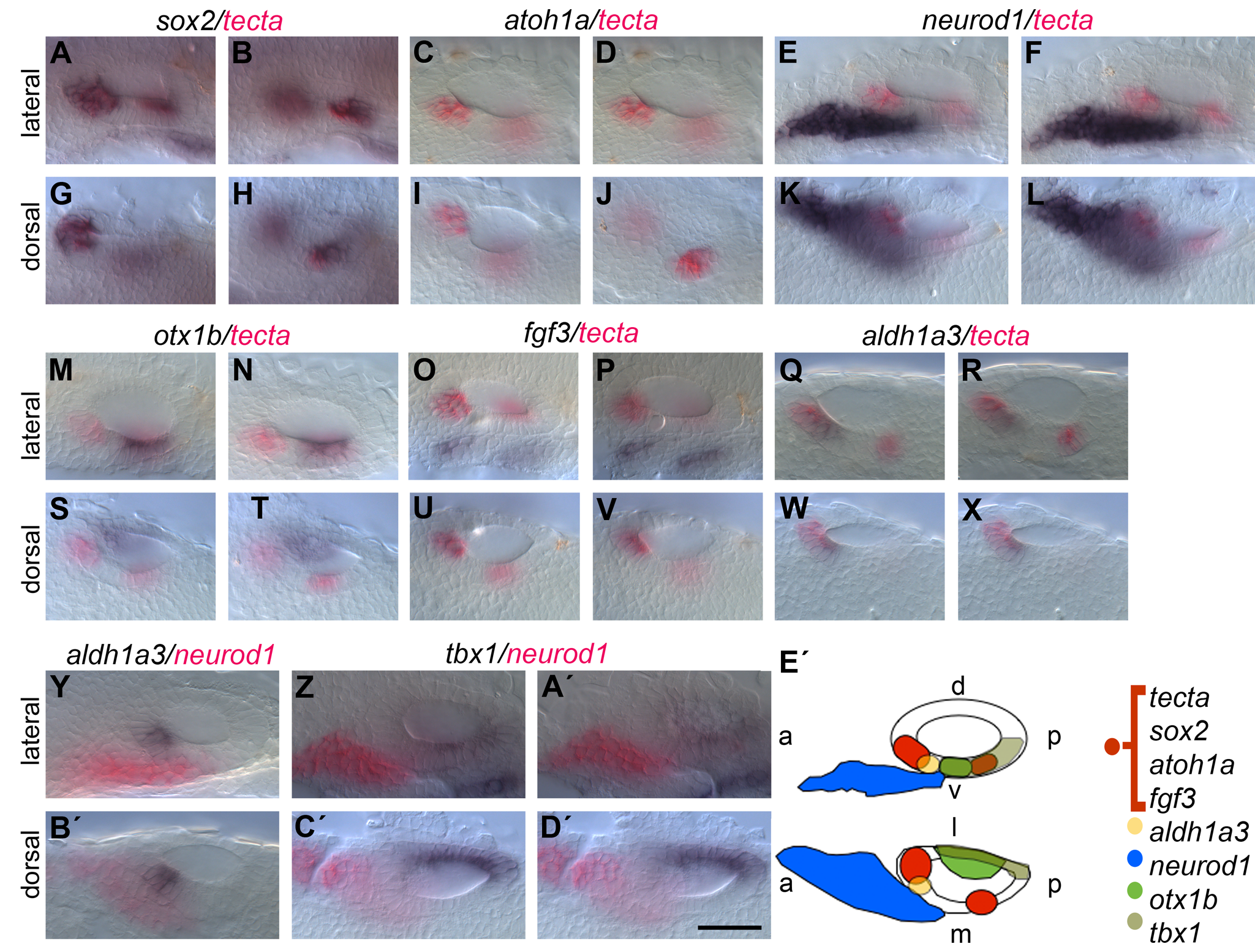

Supplement: S4 Figure — Wild-type expression pattern of sox2, atoh1a, tecta, neurod1, tbx1, otx1b, fgf3 and aldh1a3 in the zebrafish otic vesicle at 26 hpf. (A,B,G,H) sox2 (purple) and tecta (red) are co-expressed in the presumptive anterior and posterior maculae. Expression of sox2 (purple) is broader compared with expression of tecta. (C,D,I,J) atoh1a (weak purple) and tecta (red) are co-expressed in the presumptive anterior and posterior maculae. Expression of atoh1a (purple) is more restricted compared with expression of tecta. (E,F,K,L) The neurogenic marker neurod1 (purple) is mainly expressed in neuroblasts of the statoacoustic ganglion beneath the OV; expression of tecta (red) marks the developing sensory maculae in the otic epithelium. (M,N,S,T) The non-neural marker otx1b (purple) and tecta (red) are expressed in distinct domains in the OV. Expression of otx1b can be detected in a ventrolateral domain. (O,P,U,V) fgf3 (purple) and tecta (red) are co-expressed in the anterior OV. (Q,R,W,X) aldh1a3 (purple) is expressed in the anterior OV, partially overlapping with the expression domain of tecta (red) but extending more ventromedially. (Y,B′) aldh1a3 (purple) is expressed in the anterior OV in a position next to the expression domain of neurod1 (red). (Z,A′,C′,D′) tbx1 (purple) is expressed in the ventrolateral OV, posterior to the expression domain of neurod1 (red). (E′) Schematic representation of the expression domains in relation to each other. a: anterior, p: posterior, d: dorsal, v: ventral, l: lateral, m: medial. Scale bar: 50 µm. (TIF) [file pgen.1004858.s004.tif]

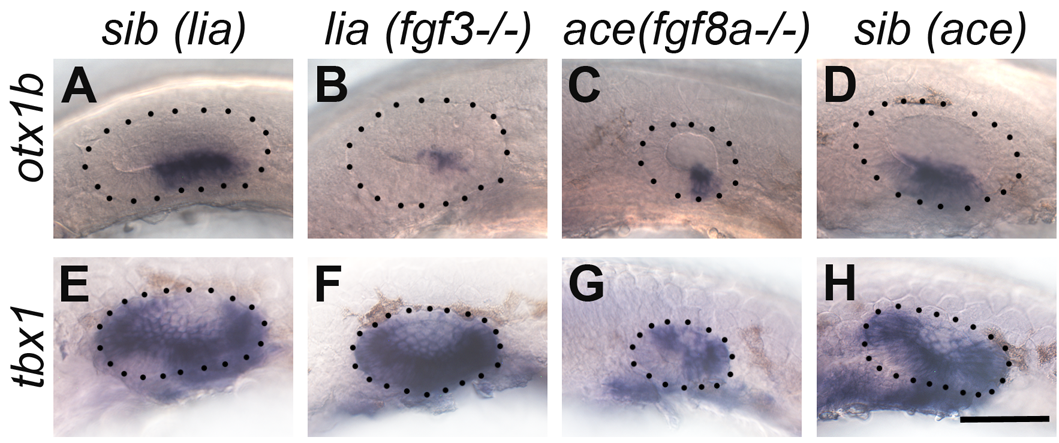

Supplement: S5 Figure — Expression of otx1b and tbx1 in lia (fgf3−/−) and ace (fgf8−/−) mutant embryos. (A, D) Expression of otx1b is normal in lia sibling (n = 65/85 embryos from a heterozygous cross) and ace sibling (n = 64/91) embryos. (B) The expression domain and levels of otx1b are reduced in the lia mutant otic vesicle (n = 20/85). (C) The expression domain of otxb1 is reduced in the much smaller ear of ace mutant (n = 27/91) embryos, but the overall pattern and level are normal. (E, H) Expression of tbx1 is normal in lia sibling (n = 64/87) and ace sibling (n = 59/82) embryos. (F) Expression of tbx1 is expanded anteroventrally in the OV of lia mutant (n = 23/87) embryos, filling in the anteroventral zone that is normally free of tbx1 expression. (G) The pattern of tbx1 expression is almost normal in ace mutant (n = 23/82) embryos, despite the smaller size of the OV. All panels are lateral views with anterior to the left. Scale bar: 50 µm. (TIF) [file pgen.1004858.s005.tif]

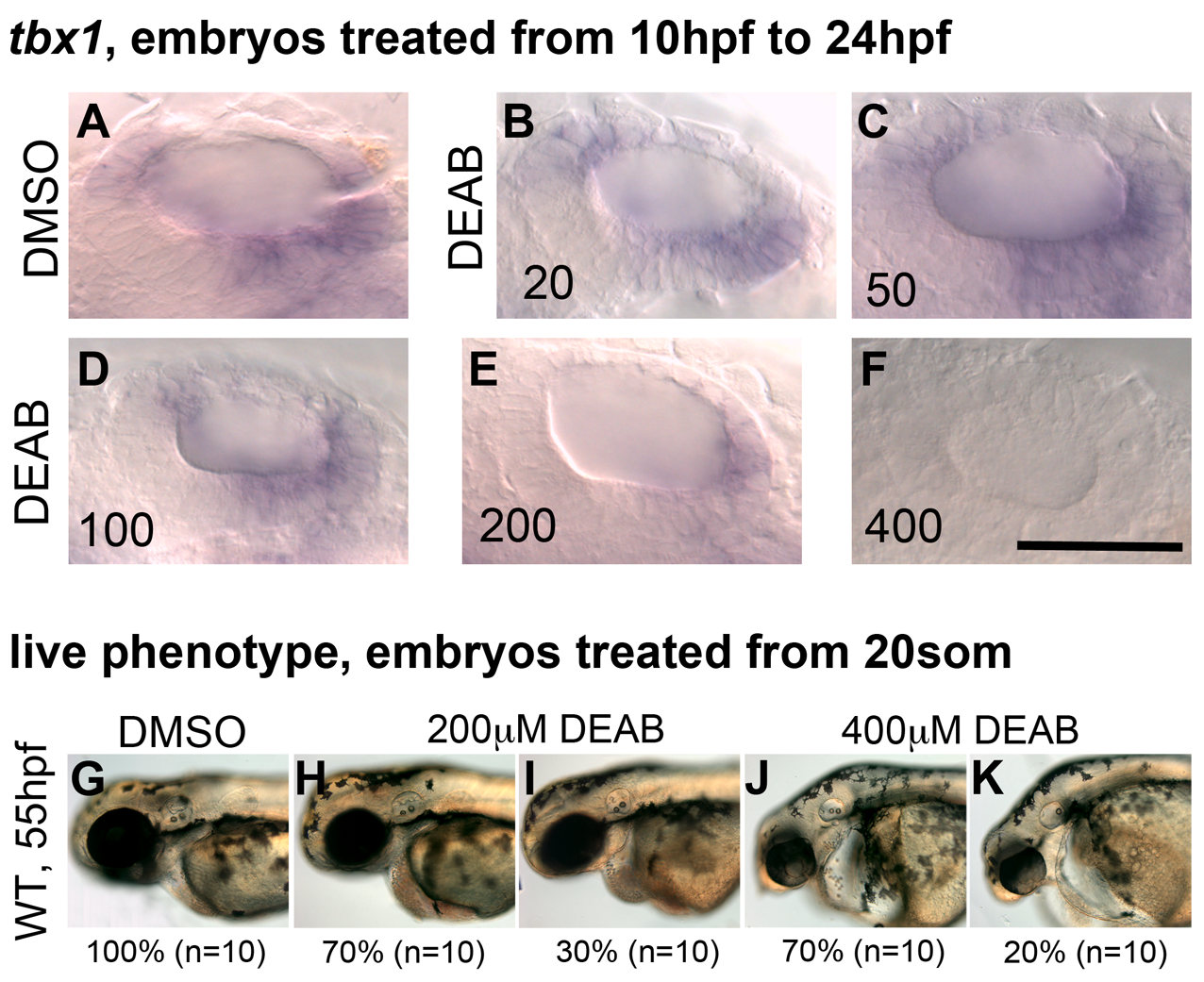

Supplement: S6 Figure — Titration of the RA inhibitor DEAB. Note: this experiment repeats published data [2] where DEAB treatment from 10 hpf to 24 hpf resulted in a complete loss of otic tbx1 expression. (A–F) Embryos were treated from 10 hpf to 24 hpf with DMSO (A) or varying concentrations of DEAB (B–F) and stained for tbx1 at 24 hpf. (A) DMSO-treated embryos display a normal pattern of tbx1 expression (n = 10). (B–D) No change in tbx1 expression is detected in embryos treated with 20 µM (B, n = 62), 50 µM (C, n = 35) or 100 µM (D, n = 11) DEAB. (E) Expression of tbx1 is down-regulated but not completely blocked in embryos treated with 200 µM DEAB (n = 13). (F) tbx1 expression is blocked altogether in embryos treated with 400 µM DEAB (n = 14). (G–K) WT embryos treated with DMSO (G) or DEAB (H–K) at 18/20S, washed at 26 hpf and grown on to 55 hpf. (G) WT embryos treated with DMSO develop normally. (H,I) In WT embryos treated with 200 µM DEAB, ear development is fairly normal (H) or slightly perturbed (I). (J,K) In WT embryos treated with 400 µM DEAB, ear development is severely perturbed in most cases (90%; n = 9/10). The head is also reduced in size and embryos display pericardial oedema. All panels are lateral views with anterior to the left. Scale bar: 50 µm. (TIF) [file pgen.1004858.s006.tif]

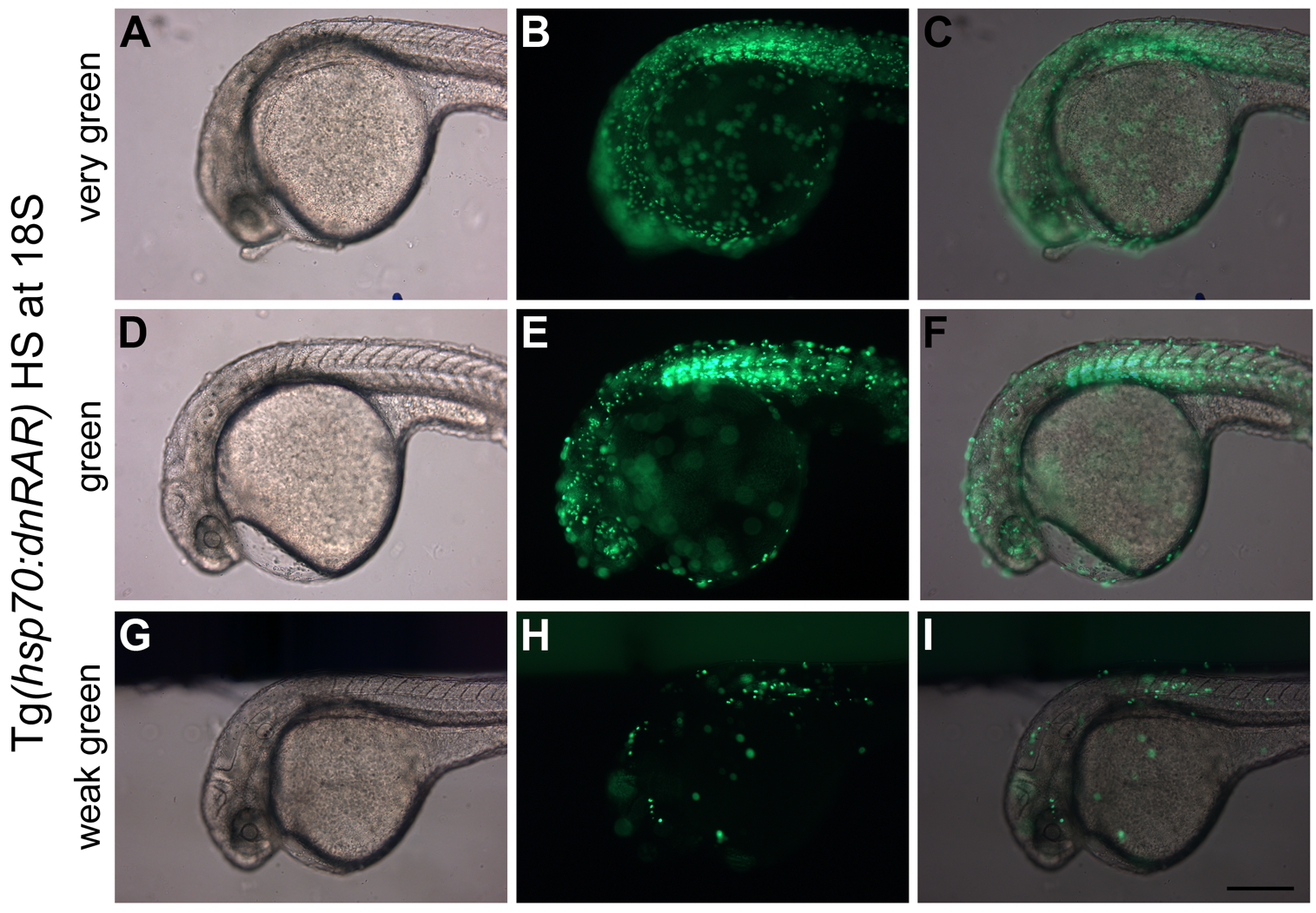

Supplement: S7 Figure — RA-inhibition: mosaic expression of GFP in Tg(hsp70:dnRAR) embryos and phenotype of DEAB-treated embryos at 55 hpf. (A–I) Embryos were heat shocked from 18S and pictures were taken at 26 hpf of embryos from one clutch of Tg(hsp70:dnRAR)♂ × nacre ♀. In all embryos, expression was mosaic in the otic region; representative embryos classified as ‘very green’, ‘green’ and ‘weak green’ are shown. All panels are lateral views with anterior to the left. Scale bar: 250 µm. (TIF) [file pgen.1004858.s007.tif]

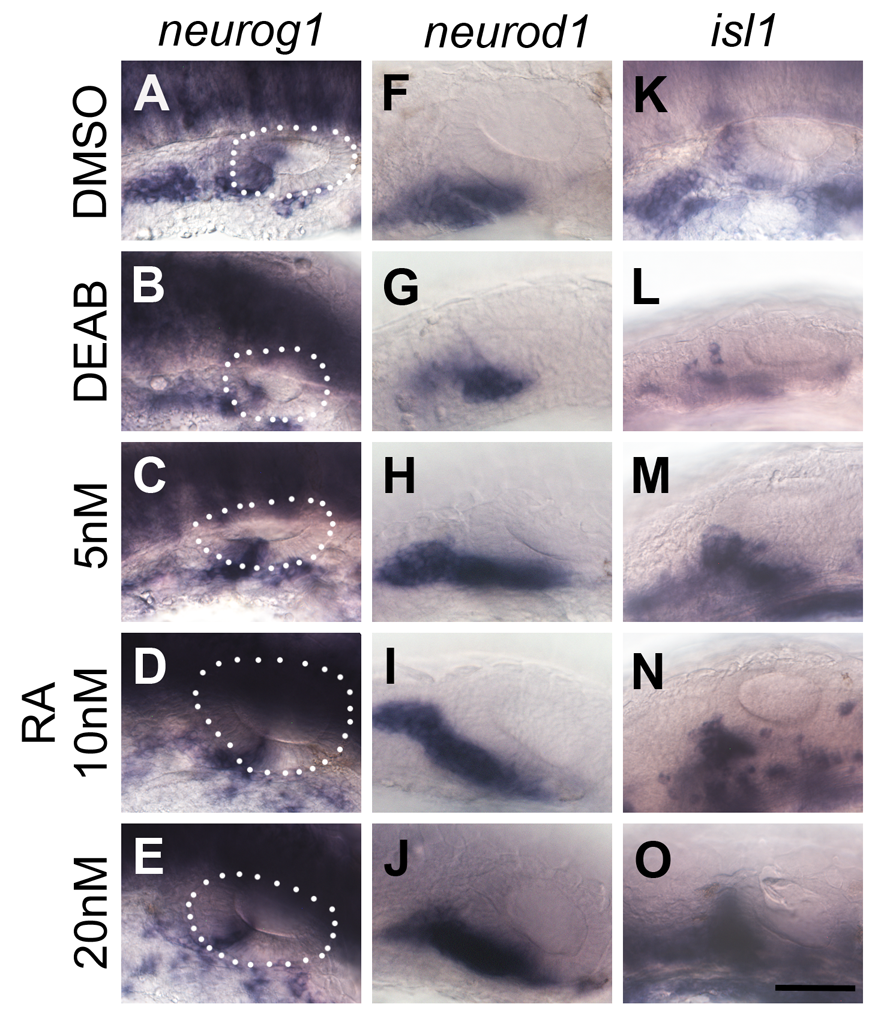

Supplement: S8 Figure — A role for RA in regulating zebrafish otic neurogenesis. Embryos were treated with DMSO, DEAB or RA from 18/20S to 26 hpf. (A–E) The dotted line demarcates the OV. Wild-type embryos treated with DMSO show normal expression of neurog1 (A), while expression is decreased in the OV of wild-type embryos treated with DEAB (B). Otic expression of neurog1 is relatively normal, or slightly reduced, in embryos treated with 5 nM (C), 10 nM RA (D) and 15 nM (E) RA. (F–J) Embryos treated with DMSO show normal expression of neurod1 (F), while expression is decreased in the OV of embryos treated with DEAB (G), and increased in embryos treated with 5 nM (H), 10 nM (I) and 15 nM (J) RA. (K–O) Embryos treated with DMSO show normal otic expression of isl1 (K), while expression is decreased in embryos treated with DEAB (L), and increased in embryos treated with 5 nM (M), 10 nM (N) and 15 nM (O) RA. All panels are lateral views with anterior to the left. Scale bar: 50 µm. (TIF) [file pgen.1004858.s008.tif]
